# Chitosan-coated mesoporous MIL-100(Fe) nanoparticles as improved bio-compatible oral nanocarriers

**DOI:** 10.1038/srep43099

**Published:** 2017-03-03

**Authors:** T. Hidalgo, M. Giménez-Marqués, E. Bellido, J. Avila, M. C. Asensio, F. Salles, M. V. Lozano, M. Guillevic, R. Simón-Vázquez, A. González-Fernández, C. Serre, M. J. Alonso, P. Horcajada

**Affiliations:** 1 Institut Lavoisier, CNRS UMR 8180, Université de Versailles Saint-Quentin-en-Yvelines, 45 Av. des Etats-Unis, 78035 Versailles cedex, University Paris-Saclay, France; 2 Synchrotron SOLEIL & Université Paris-Saclay, L’Orme des Merisiers, Saint-Aubin - BP48, 91192 Gif-sur-Yvette Cedex, France; 3 ICGM - UMR5253- Equipe AIME, Université Montpellier II, 2 Place Eugène Bataillon - CC 1502, 34095 Montpellier CEDEX 5, France; 4 Immunology, Biomedical Research Center (CINBIO) and Institute of Biomedical Research of Vigo (IBIV), Universidad de Vigo, Campus Lagoas Marcosende, 36310 Vigo, Pontevedra, Spain; 5 Nanobiofar. Center for Molecular Medicine and Chronic Diseases (CIMUS), Universidad de Santiago de Compostela, Av. Barcelona s/n, Campus Vida, 15706 Santiago de Compostela, Spain; 6 IMDEA Energy, Av. Ramón de la Sagra 3, 28935 Móstoles, Madrid, Spain

## Abstract

Nanometric biocompatible Metal-Organic Frameworks (nanoMOFs) are promising candidates for drug delivery. Up to now, most studies have targeted the intravenous route, related to pain and severe complications; whereas nanoMOFs for oral administration, a commonly used non-invasive and simpler route, remains however unexplored. We propose here the biofriendly preparation of a suitable oral nanocarrier based on the benchmarked biocompatible mesoporous iron(III) trimesate nanoparticles coated with the bioadhesive polysaccharide chitosan (CS). This method does not hamper the textural/structural properties and the sorption/release abilities of the nanoMOFs upon surface engineering. The interaction between the CS and the nanoparticles has been characterized through a combination of high resolution soft X-ray absorption and computing simulation, while the positive impact of the coating on the colloidal and chemical stability under oral simulated conditions is here demonstrated. Finally, the intestinal barrier bypass capability and biocompatibility of CS-coated nanoMOF have been assessed *in vitro*, leading to an increased intestinal permeability with respect to the non-coated material, maintaining an optimal biocompatibility. In conclusion, the preservation of the interesting physicochemical features of the CS-coated nanoMOF and their adapted colloidal stability and progressive biodegradation, together with their improved intestinal barrier bypass, make these nanoparticles a promising oral nanocarrier.

Among the different drug nanocarriers proposed so far (*e.g*. lipids, polymers, metal clusters, carbon structures, inorganic oxides), nanometric porous Metal-Organic Frameworks (nanoMOFs) have recently attracted a great deal of attention owing to their large porosity and versatile composition and topology, enabling remarkable loadings of a large variety of active molecules (drugs, cosmetics, biological gases, etc.) together with their progressive releases under physiological conditions^1^,^2^. The vast majority of the investigations dealing with drug delivery applications of nanoMOFs focus in the intravenous administration, albeit the oral route is one of the most commonly-used due to its non-invasive nature and the fact that avoids patient pain and discomfort. Even with these clear advantages, the oral route also presents some limitations in the administration of certain molecules, including inadequate intestinal absorption, poor stability to the aggressive gastrointestinal conditions or solubility issues (*e.g*. peptides, antibiotics)^3^. To circumvent these issues, some of us reported for the first time tablets, compatible with an oral administration, based on micrometric iron(III) carboxylate MOFs (MIL-53 and MIL-100; MIL stands for Material of Institut Lavoisier) loaded with important amounts of ibuprofen^4^. However, to the best of our knowledge, no oral nanoscaled MOF devices bioadapted to the drug administration have been reported so far. These systems could improve the intestinal absorption of active ingredients (AIs) via a local controlled release near to the intestinal mucosa or even, crossing the membrane while carrying the AIs in their porosity.

Recent advances in nanomedicine have evidenced that the effectiveness of drug administration is strongly affected by the design of an appropriate carrier and/or formulation^5^,^6^. Physicochemical properties (*i.e*. particle size, surface charge, rheological properties, colloidal stability) of drug nanocarriers will determine their affinity with different biological structures, their biodistribution and therefore, their efficacy. Hence, the actual challenge for the use of nanoparticles (NPs) in oral administration resides not only on the proper control of the particle size and stability but also in their suitable multifunctional design. The efficient surface engineering of nanoMOFs may contribute to reach this aim by: i) improving the chemical and colloidal stability in physiological media and/or allowing more complex specific formulations, ii) enhancing bioactivity, iii) tuning their *in vivo* fate, recognition capabilities and biocompatibility^7^ and/or iv) introducing additional biological functionalities such as imaging or sensing^8^,^9^.

Some examples of surface modification strategies of porous nanoMOFs have been reported to date^10^,^11^,^12^,^13^,^14^,^15^,^16^,^17^. However, as initially evidenced by the nanoMOF functionalization with poly(ethylene glycol) chains^10^ the challenge still remains in the specific modification of the external surface, avoiding the intrusion of the polymer (or biomolecule) within the pores of the nanoMOF, which is associated with a reduction of the MOF porosity, thus preventing its loading/release abilities. Lin *et al*. reported a strategy to improve the colloidal and chemical stability of a porous Ln-based^11^ and an iron carboxylate nanoMOF^12^, that consisted in coating the particles with a silica layer, although no information about their structural and textural properties was provided. Another example by Mirkin *et al*.^13^ used a click reaction of the UiO-66-N_3_ NPs with functionalized DNA, which permitted to enhance cellular transfection capabilities, avoiding the DNA insertion into the pores by size exclusion. In this line, some of us also proposed an efficient green one-pot surface engineering approach using biopolymers. In particular, the external functionalization of iron(III) trimesate NPs with biocompatible cyclodextrins (CDs)^14^ or heparin^15^ led to a strong improvement of the colloidal stability in body fluids, whereas maintained intact their porosity, crystallinity and encapsulation/release abilities. Furthermore, these brought to the nanoMOFs advanced functionalities suitable for the intravenous administration, allowing an increase of nanoMOF interactions with specific receptors (targeting) and/or the escape from the immune system, associated with longer circulation times (stealth).

The present work aims to prepare for the first time a suitable oral MOF nanocarrier by selectively engineering its outer surface with the biopolymer chitosan (CS). This biocompatible polysaccharide may protect the NPs against enzymatic degradation while provides them with bioadhesion properties, which is suitable for a potential local release of the AI cargo near to the intestinal mucosa. In addition, CS coating could *a priori* facilitate the transport of AI-containing MOF nanocarriers across mucosal barriers, improving the AI absorption^18^,^19^. The MIL-100(Fe) material in the form of NPs has been selected due to (i) its easy and biofriendly multigram-synthesis at the nanoscale^20^,^21^, (ii) its lack of *in vivo* toxicity^22^, (iii) its exceptional loading of challenging drugs, together with their controlled release under physiological conditions, (iv) its interesting imaging properties^10^ and (v) the potential tuning of its biodistribution by the functionalization of its external surface^14^,^15^.

The resulting CS-coated MIL-100(Fe) NPs have been fully characterized, with particular attention to their structural, chemical and colloidal stability under physiological oral conditions. In addition, the specific interactions between the CS and the MIL-100(Fe) NPs have been investigated using a combination of high-resolution X-ray Absorption Near-Edge Structure (XANES) and computing simulation techniques, approaches almost unexplored on biopolymer-decorated NPs. Finally, the biocompatibility of these coated NPs and their ability to bypass the intestinal barrier have been also evaluated under *in vitro* conditions.

## Results and Discussion

### Preparation of CS_MIL-100(Fe) NPs and physicochemical characterization

The coating of the outer surface of MIL-100(Fe) NPs with CS is based on a fast and simple impregnation method recently developed for the heparin polymer by some of us^15^ with some specific modifications. A suspension of CS (32 mg) in 7 mL of water was added to a suspension of MIL-100(Fe) NPs (30 mg) in 6 mL of ethanol. This MIL-100:CS stoichiometry (72:1 molar relation) was selected as the optimal ratio from a series of synthesis using different amounts of CS, since it provided the maximal coverage in the absence of co-precipitation (see experimental details in Methods). Attempts to improve the degree of coating by increasing the initial amount of CS resulted unsuccessful due to co-precipitation. The use of 45% (v/v) mixture of ethanol and water as the reaction medium was preferred from a variety of different solvents such as AcOH 1%, water and ethanol, since fulfills both requisites, the appropriate dispersion of the NPs and the solubilization of CS, without compromising the NP-CS interaction. The successful grafting and preservation of the main features of the porous structure in the CS_MIL-100(Fe) NPs were monitored through a set of experimental techniques.

Analysis of the particle size by dynamic light scattering (DLS) was performed in water before and after surface modification, affording a mean size distribution of 135 ± 20 and 204 ± 32 nm, respectively. The increase of around 70 nm in the hydrodynamic diameter of the NPs after the CS functionalization suggests the presence of an external corona of *ca*. 35 nm in thickness. TEM images show no morphological changes before and after the CS coating (Figure S2). A slightly visible external layer of around 20 nm can be distinguished with a highly carbonaceous composition (>20 times higher than the inside), consistent with a CS layer in a dry state.

The analysis of ζ-potential is an extremely valuable characterization to describe the effectiveness of an external chemical modification, since a change on the NP surface may provoke substantial differences in the observed charge surface. The drastic change from negative to positive ζ-potential values before and after external functionalization (−18 ± 5 and +38 ± 5 mV, respectively; see Table 1) clearly supports the presence of CS moieties coating the surface of the NPs, in agreement with the presence of the protonated amino groups (pka∼6.5) of the CS (see Figure S5).

The extent of CS coating was estimated by fluorescence spectrometry after impregnation of the NPs with a Rh-labeled CS. This quantification was performed based on three independent syntheses (scale-down x10 times). Evaluation of all the supernatants, both from the reaction mixture and washing steps, led to a 49.1 ± 0.3 wt % content of CS in the coated NP (more details in SI). This corresponds to a high efficiency of the CS grafting of 94.4 ± 0.4% after only 30 min. of contact time (considering that 100% = the total amount of CS in the initial impregnation conditions). These values were also confirmed by direct quantification of the supernatant of Rh-CS-NPs upon degradation in NaOH_(aq)_ (1 M)^23^. Thermogravimetric analysis (TGA) of the CS-coated NPs displayed a weight loss of *ca*. 38 ± 10 wt % (based on 5 independent synthesis), which further confirms the fluorescence results (Figure S1). Fourier transform infrared (FT-IR) spectra of the MIL-100(Fe) NPs, CS-coated material and commercial CS are depicted for comparison in Figure S2. In the coated material, the main absorption bands of both, the carboxylate groups in MIL-100(Fe) (1577 and 1450 cm^−1^) and the saccharide structure (1164, 1092 and 1042 cm^−1^) can be distinguished, confirming the presence of the CS on the NPs, even though the amide bands of the CS (1662, 1605 and 1393 cm^−1^) are not detected, which may be due to an overlap with the carboxylate bands of MIL-100(Fe). Finally, X ray powder diffraction (XRPD) analysis established that the crystallinity of MIL-100(Fe) NPs remains unaltered after surface modification (Figure S3), although a typical peak broadening is observed for the nanoMOFs due to the reduced crystallite size. N_2_ sorption isotherms before and after coating (Figure S4) led to comparable Brunauer-Emmett-Teller (BET) surface areas of 1570 ± 50 and 1590 ± 40 m^2^ g^−1^, respectively (note that for the CS-coated NPs a weight correction is applied considering the amount of grafted CS). Notably, this confirms that the sorption capacity of the NPs is kept after coating, in agreement with the presence of CS molecules lying only at the outer surface of the NPs and not blocking the pores of the nanoMOF.

Considering that all the CS molecules are located on the external surface of the NP, it can be estimated that an average of 17100 CS chains are coating each single NP, which is consistent with a surface density of 0.3 CS chain·nm^−2^. One can also estimate the polymer density using the conformation of the polymer chains on the NP external surface (see Supporting Information for details). By analysis of the Flory radius, we estimate that CS chains are expected to follow a “brush” conformation with partial folding of the polymer.

**Figure 1:**
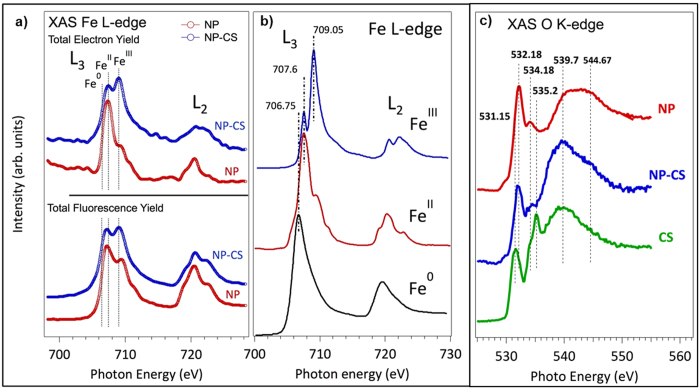
(**a**) Comparison of TFY and TEY Fe L_2,3_-edge XANES of the nano-MOFs before (NP) and after (NP-CS) CS absorption. (**b**) Reference Fe L_2,3_-edge XANES in three different model compounds with different number of *d*–electrons, coordination numbers and with different formal charges, *i.e*. metal (Fe^0^), FeCl_2_ (Fe^II^) and Fe_2_O_3_ (Fe^III^). (**c**) XANES O K-edge spectra of the nano-MOFs (NP)- before and after CS absorption, using TFY detector.

With the aim of providing insights into the chemical nature of the CS-MOF interaction, we have determined the electronic structure of the nanoMOF before and after CS functionalization by means of high-resolution soft X-ray spectroscopy, carried out at the Synchrotron Source SOLEIL. In particular, XANES spectra of the Fe L-edge and the O K-edge have been analyzed (Fig. 1)^24^,^25^. This quantitative technique permits not only to reveal the chemical functionality involved in the CS-MOF interaction but also determines to which extent this modifies the chemical nature of the NPs and the CS. Figure 1a shows the Fe L-edge absorption spectra of non-coated and CS-coated MIL-100(Fe) NPs recorded using Total Electron Yield (TEY) and Total Fluorescence Yield (TFY) simultaneous detection with superficial (*ca*. 2 nm) and bulk (*ca*. 500 nm *vs*. NP diameter of around 150 nm) probe depth, respectively. The experimental spectrum obtained for the uncoated MIL-100(Fe) in extreme surface sensitivity detection mode shows almost exclusively reduced Fe^II^ species at the upmost surface layer, whereas the spectrum recorded with fluorescent detector (corresponding to the porous bulk of the NPs), shows a ratio Fe^II^:Fe^III^ of 2:1. This is at first sight different from our previous results, showing less than one iron *per* trimer reduced under vacuum^26^. The higher observed Fe^II^ content is here probably associated with mostly surface species, which possess a slightly different local environment than bulk species, although one cannot exclude that under the beam a deeper reduction of iron species occurs.

A significant difference can be observed for the CS-coated MIL-100(Fe) NPs, with a considerable increase of the characteristic Fe^III^ peak. The more external surface of the nanoMOF is characterized by an average composition of 60% of Fe^II^ and 40% of Fe^III^, while the bulk region, which comprises the whole NP, shows a concentration of 50% of Fe^II^ and 50% of Fe^III^. This observation strongly supports an electronic stabilizing effect of the CS coating over the Fe^III^ species, and strengthens the idea of an effective interaction between the Fe at the surface of the NP and the CS coating.

CS comprises a family of N-acetylated chitins, deacetylated to different degrees (Figures S5 and S6). The degree of polymerization and N-acetylation (da) are two important parameters influencing their biomedical activity^27^. Interestingly, using NEXAFS O K-edge spectra, we can individually identify the CS functional groups interacting with the MIL-100(Fe) surface. Although the chemical structure of chitin and CS is quite similar the chemical reactions they undergo are often rather distinctive. Both polymers possess reactive hydroxyl and amino groups. In the O K-edge spectra (Fig. 1c) both the amide and the alcoholic groups can be clearly recorded by individual and well distinctive peaks. Thus, the oxygen XANES spectra show the fine spectroscopic signature of different oxygen corresponding to the specific functional groups of the CS (O-H, C=O(NH_2_) and C-O-C) before and after the interaction with the NPs. In particular, the bands at 532.2 and 535.6 eV can be assigned to the 1 s →  π * (C=O) transition of the amide^28^, and to the 1 s → σ * (C − O) transition from the alcoholic groups^29^,^30^,^31^, respectively. Importantly, working with the extreme surface probe we have clearly observed the disappearance of the band assigned to the hydroxyl groups after the CS-grafting, while no significant perturbation is detected in the band assigned to the amine functionality. This strongly suggests that the main interaction between the CS and the NPs is established via the hydroxyl groups of the CS polymer and the superficial Fe species of the NPs.

**Figure 2:**
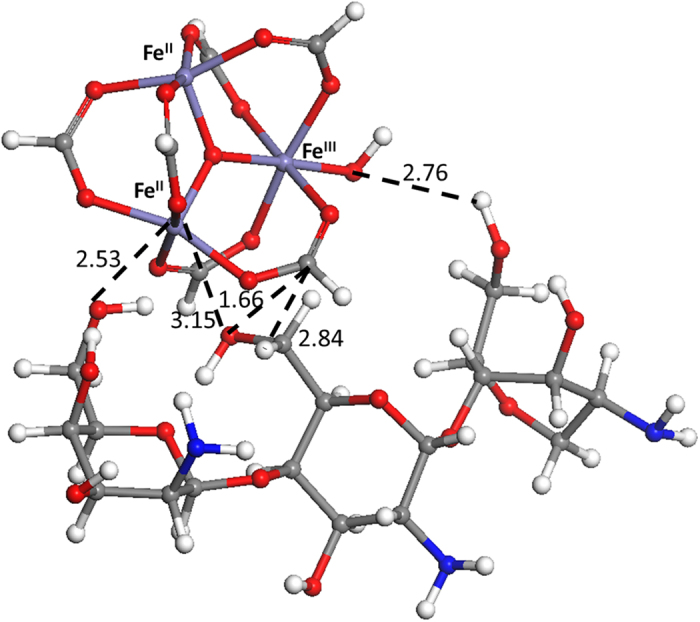
Geometry optimized configuration for CS (here corresponding to 3 units linked through β-(1–4) bridges: 2 units of D-glucosamine and 1 of N-acetyl-D-glucosamine) and a metal cluster issued from MIL-100(Fe) NPs with 2 Fe^II^ and 1 Fe^III^.

To shed more light on the potential interactions between CS and the nanoMOF surface, Density Functional Theory (DFT) molecular simulations have been performed using a simple approximation without taking into account the iron spin state. For that purpose and considering previous results from XANES, metal trimers containing 2 Fe^II^/1 Fe^III^ have been geometrically optimized in presence of a CS monomer built from 3 units linked through *β*-(1–4) bridges: 2 units of D-glucosamine and 1 of N-acetyl-D-glucosamine (Figure S5). Interestingly, both Fe^II^ and Fe^III^ sites are able to interact with the CS. If Fe^II^ strongly interacts by direct interaction with the OH groups in C3 (*d*(Fe^II^-O) = 2.5 Å) and C6 (*d*(Fe^II^-O) = 3.1 Å) of the D-glucosamine unit (Figs 2 and S5), Fe^III^ is able to indirectly interact by the formation of a hydrogen bonding between the oxygen of its coordinated OH group and the proton of the HO-C6 (2.7 Å) of the D-glucosamine unit (Fig. 2). These results are in agreement with previous observation by XANES, reinforcing the existence of strong interactions between the MIL-100(Fe) NPs and the hydroxyl groups of CS. In addition, the interaction of CS with the Fe^III^ *via* its coordinated OH group could avoid the reduction of iron, stabilizing the oxidized form.

### Chemical and colloidal stability studies

One of the main advantages of surface modification lies in its capacity to confer enhanced chemical and/or colloidal stability to the NPs. In other words, an appropriate oral grafting is expected to be chemically robust in the desired physiological gastrointestinal media for a considerable time. The CS modification may therefore alter the specific oral chemical stability of MIL-100(Fe) NPs, as it has been already demonstrated with heparin coated NPs under simulated intravenous conditions^14^. The chemical stability of the CS coated NPs has been separately investigated through i) monitorization of the release of CS molecules by using a fluorescent-labelled CS and ii) the degradation profile of coated and non-coated NPs by quantifying the delivery of the organic linker (BTC).

**Figure 3:**
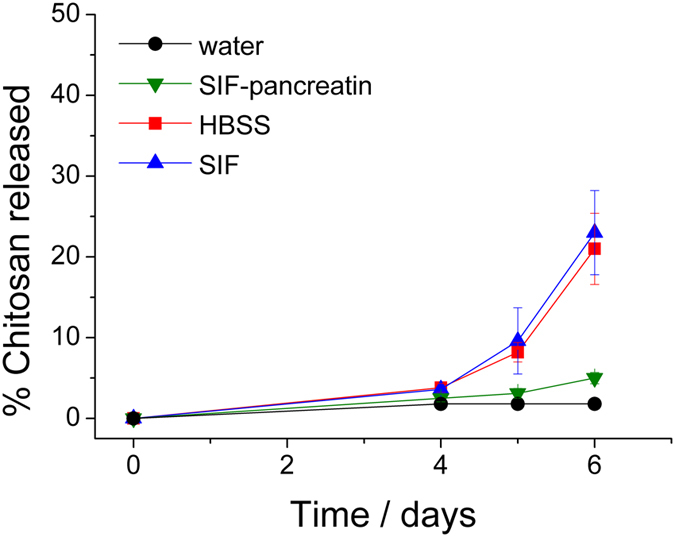
Release of Rh-CS in water (black), SIF (blue), *lis*-SIF-panc (green) and HBSS (red) at 37 °C as a function of time. The maximum of release of 100 wt% corresponds to the total release of the total amount of CS initially coating the NPs.

CS_MIL-100(Fe) and MIL-100(Fe) NPs were separately suspended in different physiological media at 37 °C, from simple pure water to more complex simulated oral conditions such as simulated intestinal media (SIF) simulating the inorganic composition of the intestine or the diluted low-ionic-strength (*lis)* SIF (dilution 1/25) supplemented with pancreatin (*lis*-SIF-panc), containing a mixture of digestive enzymes –amylases, lipases and proteases– secreted by pancreas to the intestine (see Supporting Information for media specifications; Table S1). Moreover, in view of the *in vitro* tests described later, the stability was also addressed in cell culture media such as DMEM (Dulbecco’s Modified Eagle’s medium) and HBSS (Hanks’ Balanced Salt solution) which is a high concentrated saline solution used to keep the osmotic pressure and pH in cells. In particular, the stability of the coating was investigated by monitoring the release of Rh-CS coated material in water, HBSS, SIF (which is more aggressive and therefore more convenient in this case than the diluted *lis*-SIF media) and *lis-*SIF-panc at 37 °C up to 6 days (See Methods). In the case of the CS_MIL-100(Fe) incubated in water (pH = 6.0), the release of CS was negligible up to 48 h and remained low (<2%) after 6 days, which might be directly related to the high stability of CS coating but also to the poor solubility of CS in water^32^. For the other media, HBSS, SIF and *lis*-SIF-panc, different stabilities profiles were observed. Although affording in all cases values of CS release lower than 4% after 4 days (see Fig. 3), the stability profile of CS in HBSS and SIF similarly increased up to 21 and 23% after 6 days, respectively. This indicates that *ca*. 80% of the coating remains grafted on the NPs in SIF and HBSS over this period of time, which is considered as being large enough to complete the normal human bowel transit time (12–48 h). The CS release under these buffer solutions might be related with the presence of a high concentration of ions, able to replace the CS. CS-coating results even more stable under *lis*-SIF complemented with pancreatin (*lis*-SIF-panc), with >95% of the coating remaining grafted after 6 days. In *lis-*SIF-panc medium the characteristic negative ζ-potential of MIL-100(Fe) and the positive value in CS_MIL-100(Fe) NPs, reached neutral values (*ca*. −6 mV for both coated and uncoated NPs). This change of surface charge is likely due to the presence of proteins (or here enzymes) in the media, which not only are known to enhance the colloidal stability of MIL-100(Fe) NPs owing to the formation of a protein corona on the surface of the NP, but also prevents from the fast release of the coating moieties. The large coating stability is indicative of a robust interaction between the coating and the NPs, and dismisses the idea of simple co-precipitation.

**Figure 4:**
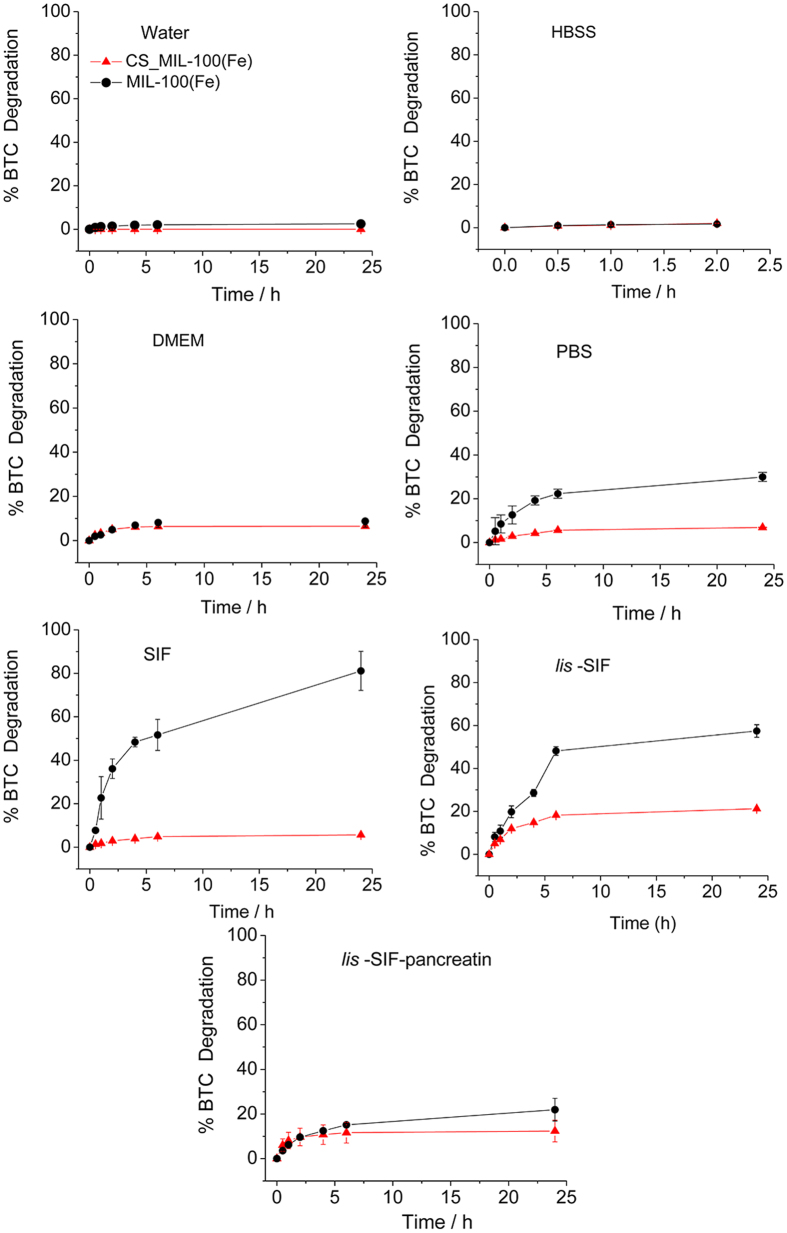
Degradation kinetics of uncoated (black) and coated (red) MIL-100(Fe) NPs in water, HBSS, DMEM, PBS, SIF, *lis*-SIF and *lis*-SIF-panc at 37 °C as a function of time.

Evaluation of nanoMOF chemical stability was then followed by the accumulative release of the organic linker (trimesic acid or BTC), quantified by HPLC (see Methods) in different media. In the case of pure aqueous solution, both uncoated and CS-coated NPs display similar degradation profiles associated with an excellent chemical stability, where less than 3% of the ligand is released after 24 h (Fig. 4). The same stability was observed using a simple solution rich in salts (HBSS), with *ca*. 2% of BTC released after 24 h (Fig. 4). In more complex media, such as cell culture media (DMEM), the BTC release profile displayed a slight increase in the NP kinetic of degradation in both, uncoated and CS-coated NPs, with *ca*. 9 and 7% of degradation, respectively. The lower stability exhibited in DMEM is related with the higher concentration of phosphate, bicarbonate and pyruvate salts, which are able to strongly bind iron and may lead to a faster degradation. Remarkably, an enhanced stability conferred by the CS coating was observed in PBS (containing a higher phosphate concentration), in which uncoated MIL-100(Fe) NPs were rapidly degraded up to 30%, whereas the CS_MIL-100(Fe) NPs led to only 7% of BTC release^33^. Such a stabilization of the CS-coated MIL-100(Fe) NPs could tentatively be assigned to a slower diffusion of the phosphates through the CS layer, which would hamper the exchange with the carboxylate ligands. In addition, other simulated intestinal complex media have been evaluated to predict the behavior of these NPs under the targeted oral conditions. After 24 h in SIF, only 6% of the BTC ligand was released for the CS-coated NPs compared with *ca*. 80% of BTC for the uncoated NPs, suggesting an adapted chemical stability of CS-coated NPs for oral administration associated with a beneficial effect of the CS-coating. When this SIF medium is diluted (*lis*-SIF) and complemented with pancreatin (*lis*-SIF-panc), the chemical stability of MIL-100(Fe) NPs intensely improves, presenting degradations that remain at *ca*. 60 and 20% of BTC release (*vs*. 80% in pure SIF) respective for *lis*-SIF and *lis*-SIF-panc, while maintaining a low degradation of *ca*. 20 and 12% (*vs*. 6% in pure SIF) respectively for *lis*-SIF and *lis*-SIF-panc in the case of CS_MIL-100(Fe) NPs (Fig. 4). Although one could *a priori* expect a lower NP chemical stability in *lis*-SIF-panc as a consequence of an enzymatic degradation, this result supports the hypothesis of the formation of a protein corona on the surface of the NP, which highly improves the chemically stability of the two materials. Therefore, we can conclude that CS-coating effectively improves the chemical stability of MIL-100(Fe) NPs in simulated physiological media. This contrasts with the results observed in previous studies developed by some of us, using heparin as the coating material, in which similar degradation profiles were exhibited regardless the heparin coating^15^, supporting the influence of the nature of the external surface agent on the chemical stability of NPs.

In a second step, the effect of the CS coating on the colloidal stability of CS-coated MIL-100(Fe) NPs was evaluated in diverse media in view of their *in vitro* test and potential oral administration and compared with the uncoated MIL-100(Fe) NPs, recently studied by some of us^33^. For this, pure water, PBS, cell culture media (DMEM and HBSS) and different intestinal physiological media, such as the *lis*-SIF, *lis*-SIF-panc (which permits to specifically evaluate the influence of pancreatin) or *lis*-SIF-muc (containing a glycosylated protein, which is the major macromolecular constituent of intestinal mucus) were selected (Figure S7 and Table S1; see Methods). When monitoring the NP size, the uncoated MIL-100(Fe) NPs exhibited a suitable colloidal stability in water up to 4 h, whereas CS-coated MIL-100(Fe) started to aggregate after 1 h (Figure S7). In case of PBS medium, both uncoated and CS-coated NPs did not show any significant aggregation up to 6 h (from 135 ± 20 nm to 155 ± 61 *vs*. 182 ± 50 nm in MIL-100(Fe) and CS_MIL-100(Fe) NPs, respectively), maintaining even a higher degree of monodispersity for the coated NPs (PdI < 0.3). This might be associated with a similar ζ-potential value (around −30 mV), being large enough to electrostatically stabilize the NPs. At longer times (>6 h), both uncoated and CS-coated NPs started to aggregate, reaching particle sizes up to 1200 and 600 nm, respectively. This destabilization is in agreement with previous data^33^, where the phosphate groups of the PBS medium lead to a progressive disruption of the NP architecture. In *lis*-SIF medium, uncoated MIL-100(Fe) NPs remained stable up to 24 h, whereas in the case of the CS-coated material a slight aggregation was rapidly observed (240 nm at 4 h). This could be related with the bioadhesive properties of the CS and with the tendency to display neutral surface charge^19^, associated with weaker electrostatic repulsion forces required to stabilize the colloids (ζ-potential values of −51 ± 1 *vs*. −5 ± 3 mV in MIL-100(Fe) and CS_MIL-100(Fe) NPs, respectively; Table 1). In addition, the apparent improved colloidal stability of both NPs in *lis*-SIF in comparison with PBS could be explained by a larger degree of erosion of the framework in PBS due to the higher phosphate content (2 *vs*. 10 mM). Finally, in the particular case of HBSS, the CS-coated material showed enhanced colloidal stability as compared with the uncoated NPs (735 ± 38 *vs*. 431 ± 8 nm in case of in MIL-100(Fe) and CS_MIL-100(Fe) NPs, respectively; Table 1), indicating a shielding effect of CS, which prevents from a fast degradation and coalescence of the NPs. In the case of protein-containing media closer to real physiological intestinal conditions, *lis*-SIF-panc and *lis*-SIF-muc, the CS_MIL-100(Fe) NPs displayed poor colloidal stability with a tendency to aggregate faster than the non-coated material (168 ± 45 *vs*. 908 ± 36 nm in *lis-*SIF-panc, and 254 ± 40 *vs*. 478 ± 85 in *lis-*SIF-muc from MIL-100(Fe) and CS_MIL-100(Fe) NPs, respectively). Similarly, this aggregation effect might be explained by the absence of electrostatic repulsions associated with neutral ζ-potential values (−7 ± 2 *vs*. −6 ± 3 in *lis-*SIF-panc and −6 ± 1 *vs*. −13 ± 9 mV in *lis-*SIF-muc for MIL-100(Fe) and CS_MIL-100(Fe) NPs, respectively). In addition, the potential mucoadhesive effect of mucin and the bioadhesion of CS and/or the possible enzymatic effect, promoting the destabilization of the nanoMOF structure, might also affect the colloidal stability. A similar larger aggregation tendency was observed in DMEM (273 ± 21 *vs*. 146 ± 37 nm for coated and uncoated NPs, respectively), in agreement with the presence of proteins coming from the FBS-supplementation leading to insufficient electrostatic repulsion forces (ζ-potential = 8 ± 2 *vs*. −24 ± 0 mV in MIL-100(Fe) and CS_MIL-100(Fe) NPs, respectively), which induce a loss of nanoMOF colloidal stability. In general, the colloidal stability of CS_MIL-100(Fe) NPs follows similar tendencies as the uncoated NPs, with however a general faster aggregation (with the exception of HBSS) likely due to the bioadhesive properties of the coating agent^34^. Other examples in the literature of MOF NPs coated with CD-P^14^, DNA^13^ or lipids^16^ only show an improved colloidal stability in simple media (water or 0.2 M NaCl water solutions). A more detailed study of the colloidal stability under simulated physiological media is presented in the case of heparin-coated NPs^15^, albeit the lack of studies on oral fluids prevents us from a direct comparison.

### Toxicological concerns and intestinal barrier crossing

Taking into account that cationic polymers are often associated with cytotoxic effects^35^,^36^, cell viability of CS coated and non-coated NPs was evaluated prior to study the cell uptake and membrane transport assays. Remarkably, both coated and uncoated NPs exhibited a similar low cytotoxic profile (maximal inhibitory concentration (IC); IC_80_ = 800 μg mL^−1^) when incubated 24 h with human colorectal carcinoma cells (Caco-2), as confirmed by the MTT tests (Figure S8, See methods)^37^. These results are in good agreement with the absence of severe toxicity in other cell lines^38^, and with previous *in vivo* studies of MIL-100(Fe) NPs^22^, excluding any negative influence of the CS on the toxicity of the CS-coated nanocarrier. Furthermore, NPs can induce reversible or permanent conformational changes in proteins, occasionally strongly altering their functions and leading to associated cell damages, as recently shown by some of us (see Figure S9)^39^. For this purpose, the adsorption of the main plasmatic proteins (*i.e*. fibrinogen, albumin and globulins) on the nanoMOFs surfaces was monitored by fluorescence spectroscopy (see Experimental section) showing no significant shift of the aminoacid bands for all the tested protein fractions, regardless the surface coating and even at high nanoMOF concentrations (Figure S9). This suggests that the protein conformation remained unaltered, in accordance with previous results using heparin coated MIL-100(Fe) NPs^33^. Both, a low cytotoxicity and the absence of protein conformational changes support the low toxicity of CS-coated and uncoated NPs.

**Figure 5:**
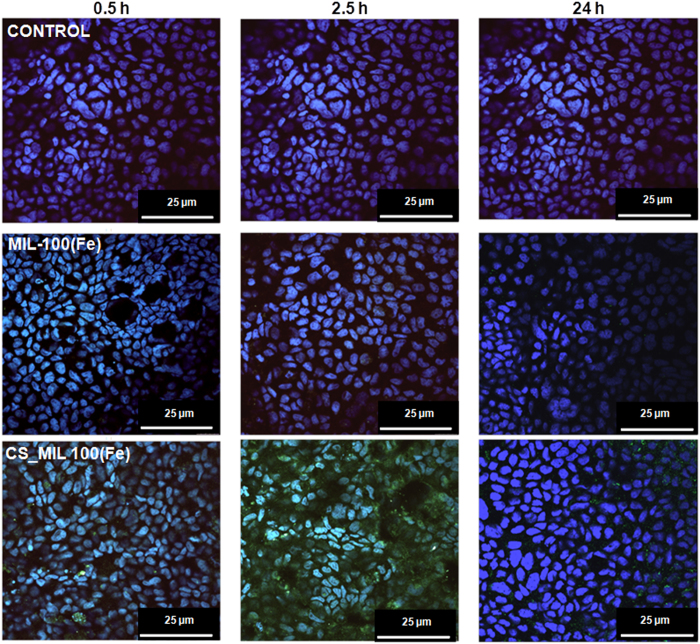
Confocal microscopy images of Caco-2 cells containing uncoated and CS-coated MIL-100(Fe) NPs observed by iron self-reflection signal (green channel) and the nucleus stained by DAPI (blue channel). The images have been taken at different times: 0.5, 2.5 and 24 h. Moreover, the controls were obtained with cells (control) after 24 h. In all the cases, the scale bar corresponded to 25 μm. All the images were taken at 63X.

In absence of cytotoxicity, the intestinal bypass of the NPs was evaluated as a function of the surface modification by using an *in vitro* model based on two chambers (donor or apical and receptor or basolateral compartments) separated by a polarized Caco-2 cells monolayer, which is widely used due to its analogy to human intestinal epithelium as well as its ability to open the epithelial tight junctions upon specific stimuli^40^. Some of us initially investigated the nanoMOF intestinal crossing by incubating the NPs with the cell monolayer in the donor (apical) chamber (0.5 and 2.5 h) and taking images on a confocal microscope at different times (0.5, 2.5 and 24 h; Figures S10–S12), directly following the iron self-reflection signal of the NPs (Fig. 5)^15^,^38^. After 2.5 h of NPs-cell monolayer contact time, the apical media was removed to eliminate the non-interacting excess of nanoMOFs and renewed with fresh cell culture media to continue cell incubation up to 24 h (see methods). Interestingly, CS_MIL-100(Fe) NPs exhibit a significantly higher cell uptake as compared with the uncoated NPs, in which only few NPs were observed (Fig. 5). This is in agreement with previous described results using CS-coated NPs of different nature^19^,^41^, in which the bioadhesive capacity of CS^34^ is associated with the increase of NPs cell penetration through an improvement of NP-cell contact^41^,^42^. To further assess whether these NPs were internalized into the cells, confocal microscopy images were collected at different levels of the Z-axis (Figures S10–S12), detecting the iron self-reflection fluorescence coming from the nanoMOFs at different depths. An improved internalization of CS-coated NPs is observed with time, with an extremely important amount of CS_MIL-100(Fe) NPs within the intestinal cells after only 2.5 h of incubation (Figs 5 and S11). The amount of NPs crossing the intestinal barrier was estimated in the basolateral compartment by HPLC-quantification of the BTC ligand (see methods; Table S3). These results indicate a higher intestinal crossing of CS-coated NPs as compared with the uncoated NPs, confirming a better cell penetration of the CS_MIL-100(Fe) NPs. After 24 h of incubation, the barrier crossing of the CS-coated NPs was 20 times larger than the crossing for the uncoated NPs (4.10 ± 0.03 *vs*. 0.20 ± 0.09%), whereas at shorter contact times the differences are less pronounced (0.44 ± 0.03% *vs*. 0.30 ± 0.02 at 30 min, and 0.50 ± 0.07 *vs*. 0.10 ± 0.04% at 2.5 h for CS-coated and uncoated NPs, respectively). Interestingly, in contrast with the slower uptake of heparin coated MIL-100(Fe) NPs by macrophages^15^, the presence of CS-coating is able to boost the cellular internalization and thus, to promote their intestinal barrier bypass. In addition, particle size was determined in the basolateral media after 24 h, observing three different populations: (i) ∼500 nm, probably corresponding to cellular debris, (ii) ∼80 nm, associated with the protein fraction present within the cell culture media and (iii) ∼200 nm, which could be related with the nanoMOFs dimensions (initially ∼200 nm and ∼100 nm for CS_MIL-100(Fe) and MIL-100(Fe), respectively). This verifies that the detection of BTC ligand within the receptor media corresponds to the bypass of intact colloidal NPs and not only to possible by-products from NP degradation.

The viability and integrity of the intestinal barrier was evaluated along with the study (24 h) in the presence of MIL-100(Fe) and CS_MIL-100(Fe) NPs by measuring the transepithelial resistance, accepted as a good model for determining cell integrity before and after the transport of chemicals^43^ (TEER; see Methods)^44^. For this purpose, the polarized *Caco-2* monolayer cell was selected as a suitable *in vitro* model, widely used due to the high homology to human intestinal epithelium, which allow investigating the oral NP absorption^45^. Importantly, TEER values remained similar to the control, supporting the lack of cell toxicity of both NPs and more interestingly, indicating that the CS coating was not able to provoke a reversible opening of the tight junctions (Figure S13), excluding the paracellular intestinal crossing *vi*a. This could be tentatively attributed to an insufficient CS amount as well as to the CS conformation, which is highly sensitive to pH variations: pH greater than 6.5 (pKa) induces a more coiled configuration of CS due to a low/zero charge density and a decrease of its aqueous solubility. At this state, CS would tend to precipitate, losing its efficiency for opening the tight junctions^46^. Considering the surface charge inversion of CS_MIL-100(Fe) NPs in HBSS and DMEM (from +18 to −24 mv, respectively; Table 1) and the absence of effect at both conditions (Figure S13), the CS amount on the surface could be insufficient to provoke the opening of tight junctions. Thus, one can rationally suggest an intestinal bypass by a transcellular route, which is partially supported by the NPs uptake within the cells. However, to clearly elucidate the mechanism of internalization of these NPs, further specific studies are required.

As it is well known, polymeric NPs can induce a systemic and mucosal immune response^7^,^47^. For this reason, the inflammatory response by the CS_MIL-100(Fe) was investigated in comparison with the uncoated MIL-100(Fe) NPs by complement activation tests and by the cytokine profile. First, the complement activation was studied by incubating different concentrations of CS-coated and uncoated NPs (25 and 250 μg·mL^−1^) in a pool of human serum (from different donors, samples obtained through an institutional ethics approval; see experimental section) and quantifying the common factor of the complement cascades C3, which is degraded in presence of specific stimulus (see methods). Remarkably, there was no induction of the complement for both NPs, regardless of the CS coating (Figure S14), dismissing the immune activation by this route. Additionally, foreign NPs such as nanoMOFs, could induce the secretion of extracellular factors involved in inflammation or immunomodulation (cytokines). Cytokines production was tested by incubating two different concentrations of the CS-coated and uncoated NPs (25 and 250 μg·mL^−1^) with human peripheral blood mononuclear cells (PBMCs) from different voluntary donors (see Experimental section). After 24 h in contact with two different concentrations of the nanoMOFs, the production of cytokines decreased from one to two orders of magnitude in the case of CS-coated NPs compared with the uncoated MIL-100(Fe) NPs (Figure S15 and Table S2), except for the interleukins IL-6 and IL-8 (involved in the pro-inflammatory response and in the antibodies production). This suggests that the CS coating reduces the immune response, which might be related with a lower NP recognition by the immune system. Indeed, decreasing the NP immune recognition is often suitable for drug delivery nanosystems since this could improve the half-life times of the nanocarriers within the body. These observations are in agreement with our previous reports that evidenced a lower macrophage recognition for heparin coated MIL-100(Fe)^15^.

## Conclusions

We have demonstrated a simple and efficient method for the selective external surface modification of MIL-100(Fe) nanoparticles based on bioadhesive chitosan (CS), that preserve both the high porous character and crystalline structure of the nanoMOF. The surface modification is associated with a *ca*. 35 nm CS layer organized into a highly-dense “brush” conformation. Combination of Fe and O XANES and DFT analysis suggested that the main interaction between CS and the outer surface of MIL-100(Fe) NPs occurs between the hydroxyl groups of the CS and terminal Fe atoms. In addition, the Fe-XANES confirms that CS-coating partially prevents Fe^III^ from its reduction, associated with a shielding effect.

The surface modification also improves chemical stability and, in some extent, colloidal stability of the nanoMOF in diverse physiological media, notably simulated physiological oral fluids. Remarkably, the CS-coating endows MIL-100(Fe) NPs with additional biological functionalities, such as an enhanced cell uptake and intestinal barrier bypass, while maintaining a high biocompatibility.

As compared with the former nanoMOF, the CS coated NPs preserve the high porous nature and crystalline structure, without raising toxicity issues. But more importantly, the CS coating confers clear advantages to the NPs that are (i) an improved chemical stability in simulated oral fluids, (ii) the introduction of an additional biological functionality such as the bioadhesive character, (iii) a superior intestinal barrier bypass, and (iv) the lower recognition by the immune system. All these features make these CS-coated NPs very promising candidates for the oral administration of active ingredients.

## Methods

### Materials

Iron(III) chloride hexahydrate (97%), 1,3,5-benzene tricaboxylic acid (95%), phosphate buffered saline (PBS) solution (0.01 M, pH = 7.4), chitosan (CS) low molecular weight (~50 kDa, 75–80% acetylation degree (DA), 200–800 cP), pancreatin from porcine pancreas, albumin from bovine serum (lyophilized powder, ≥98%), thiazolyl blue tetrazolium bromide (MTT), *p*-formaldehyde 4% (36.5–38% in H_2_O), Zymosan A from Saccharomyces cerevisiae, lectin (red kidney bean), Mowiol fluorescent mounting medium (Mowiol 4–88), and ammonium sulfate, albumin from human serum (96–99%) were purchased from Sigma-Aldrich. Rhodamine-labeled CS (5/6-carboxy-tetramethyl-rhodamine, 5/6-TAMRA) (200–500 cP, 85% DDA, degree of dye labelling substitution 1 mol %) was purchased from Creative PEGworks. Dulbecco’s Modified Eagle’s medium (DMEM) supplemented with glutamax-1, L-glutamine (2 mM), and trypsin/ethylenediamine tetra-acetic acid (Trisip-EDTA, 10 mM, pH 7.4), penicillin/streptomycin (100 U.mL^−1^), non-essential amino acids (100X) and HBSS medium (Hank’s Balanced Salt Solution) were purchased from Gibco-Life Technologies. Similarly, 2′,7′-dichlorofluorescein diacetate (2.5 μM; DCFH-DA) and Bodipy@phalloidin, 4′,6-diamidino-2-phenylindole dihydrochloride (DAPI) were provided from Life Technologies. Heat-inactivated fetal bovine serum (FBS), dimethylsulfoxide (DMSO; ≥99.7%), were purchased by Fischer. Also Millicell ERS-2 meter was purpose by Millipore, (Bedford, MA, USA), while the Transwell, Transwell cell culture chambers was distributed by Corning Costar, Cambridge, MA). Polyvinylidene difluoride membrane (PVDF, Immun-Blot, Bio-Rad; Hercules, CA), Tris Buffered Saline with Tween 20, 1x (TBS-T), 5-bromo 4-chloro 3′-indolyphosphate *p*-toluidine salt (BCIP) were provided by Bio-Rad Laboratories, Hercules, CA. Ficoll-Paque PLUS (GE Healthcare), lipopolysaccharide (LPS; InvivoGen, San Diego, CA), phorbol 12-myristate-13-acetate (PMA; Abcam, Biochemicals), Monoclonal Mouse anti-C3, C3b Antibody Human (IgG2b mouse anti- C3/C3b, Abcam-ab1187, Biochemicals) and Goat anti-Ig mouse AP (DO486, Dako) were also used. All materials were used as received without further purification.

### Synthesis of MIL-100(Fe) NPs

MIL-100(Fe) NPs (or Fe_3_OF(H_2_O)_2_(C_9_O_4_H_3_)_2_) were synthesized following a microwave-assisted hydrothermal synthesis according to a previously reported procedure^21^. Purification of MIL-100(Fe) NPs was performed as published elsewhere^33^.

### Preparation of CS coated MIL-100(Fe) NPs

30 mg of MIL-100(Fe) NPs (note: NPs were used wet, and therefore wet: dry ratio was previously determined from NPs dried at 100 °C overnight) were dispersed in 6 mL of ethanol using an ultrasound tip. In a different vial, 32 mg of CS were suspended in 7 mL of water. With a pKa value of 6.5, CS is typically insoluble in water at neutral pH, and only in more acidic solutions protonation of the amino group may occur, thus improving the solubility. Then, the two suspensions were mixed and kept under stirring for 30 min. Importantly, in these circumstances the acidic nature of the MIL-100(Fe) NP suspension (pH = 3.8) contributes to the partial protonation of the amino functionality in CS, therefore favoring its solubilization. The molar ratio between MIL-100 NPs and CS in the reaction mixture was 72:1, with MIL-100(Fe) and CS concentrations of 2.3 and 2.5 mg·mL^−1^, respectively. The CS-coated NPs were collected by centrifugation and washed with aliquots of 15 mL of AcOH 1% (v/v) (x1) and water (x5). Finally the product was stored wet in water.

### Preparation of physiological media

Simulated intestinal fluid (SIF) and *lis*-SIF (low-ionic-strength) supplemented or not with pancreatin or mucin were prepared according to the European Pharmacopeia 7.0 (see Supporting Information for details; Table S1)^48^.

### Physicochemical characterization

Fourier transform infrared (FTIR) spectra were collected using a Nicolet 6700 instrument (Thermo scientific, USA). Thermogravimetric analyses (TGA) were performed using a Perkin Elmer Diamond TGA/DTA STA 6000 (25–600 °C at 3 °C·min^−1^ with an O_2_ flow of 20 mL·min^−1^ (note that CS wt% is given with respect to dry MIL-100(Fe) weight). X-Ray powder diffraction (XRPD) patterns were collected in a D8 Advance Bruker diffractometer using Cu Kα1 radiation (λ = 1.54056 Å). Profiles were generally collected in the 3° < 2*θ* < 30° range with a typical step size of 0.02° in continuous mode. N_2_ adsorption isotherms were obtained at 77 K using a BELsorp Maxi (Bel, Japan). Samples were previously outgassed at 140 °C under vacuum for 3 h. UV-vis analysis was performed in a UV/Vis/NIR spectrometer Lambda 19 from Perkin Elmer and fluorescence measurements were collected in a FluoroMax-3 from Jobin Yvon Horiba.

### High resolution soft X-ray XANES

X-ray absorption spectra were recorded at the ANTARES beam line specially designed in performing complementary photoemission and X-ray soft absorption, at the SOLEIL Synchrotron. The ring operating conditions were of 2.5 GeV electron energy, with injection currents of 500 mA and “top-up” mode. The radiation was monochromatized using a plane-grating monochromator (PGM), which is characterized by a slit less entrance and the use of two Varied Linear Spacing (VLS) gratings with a Variable Groove Depth (VGD) along the grating lines. All measurements were performed at 20.0(2) °C over the range 670–830 eV with a step size of 0.5 eV to permit a correct normalization of the XANES spectra.

### Molecular simulation

Calculations performed on molecular clusters containing various Fe^II^:Fe^III^ ratios were performed using the PW91 GGA density functional and the double numerical basis set containing polarization functions on hydrogen atoms (DNP) as implemented in DMol^3^ code. The configurations obtained from the convergence of the calculations correspond to the global minimum and describe the plausible interactions existing between CS and the metal clusters.

### Fluorescence-based studies: Quantification of coated CS and stability of coating by CS release studies

The synthetic procedure for CS coating described above was reproduced (scale-down x10) using a rhodamine-labeled CS, which enables fluorescence quantification, and therefore determination of the amount of CS coating the NP. First, UV-visible spectra of rhodamine-labeled CS were performed in different media to determine the excitation (λ_ex_) and emission (λ_em_) wavelengths, which are respectively 552 and 574 nm. Calibration curves of rhodamine-labeled CS were obtained between an interval of 10^−2^ to 10^−5 ^mg·mL^−1^ (correlation coefficient >0.99). Fluorescence-based controlled release studies of the coated CS fluorescent probe were performed in water, SIF, *lis*-SIF-panc and HBSS at 37 °C over 6 days. In a general procedure, CS-coated NPs were dispersed in the different media at 0.2 mg·mL^−1^, while maintained at 37 °C and constant bi-dimensional stirring. The release of CS was quantified by collecting aliquots of the supernatant after centrifugation of the NP suspension at certain times. The same volume of fresh medium at 37 °C replaced these aliquots. All results are denoted as the wt% of CS released, considering as 100% of delivery the release of the total amount of CS coating the NPs.

### Quantification of trimesic acid by high performance liquid chromatography (HPLC)

Degradation kinetics of the NPs were obtained by quantifying the release of the trimesic acid (or 1,3,5-benzenetricarboxylic acid or BTC) in the different media tested (water, HBSS, DMEM, SIF, *lis*-SIF-panc) at 37 °C according to a previously reported procedure^15^. Note that the limit of quantification of trimesic acid was estimated to 0.09 μg·mL^−1^.

### Colloidal stability tests

Colloidal stability was evaluated by dynamic light scattering (DLS; Zetasizer Nano, Malvern Instruments) following the evolution of the particle size and the ζ-potential over time. NPs were dispersed by using an ultrasound tip in different media (water, *lis*-SIF, *lis*-SIF-panc, *lis*-SIF-mucin, DMEM and HBSS). Note here that for evaluating the colloidal stability in the prepared media, double concentrations have to be considered, *i.e*. an aqueous suspension of the uncoated or CS-coated NPs at 0.2 mg·mL^−1^ was added to each medium prepared at a double concentration, thus ending with the desired final concentration of 0.1 mg·mL^−1^ for the NPs and the corresponding *lis*-SIF, *lis*-SIF-panc, *lis*-SIF-mucin, DMEM and HBSS^33^.

### *In vitro* cell studies

#### Cells and culture

Human colon carcinoma Caco-2 cell line (ATCC HTB-37™) was maintained in DMEM medium supplemented with glutamax-1 with 10% of heated-inactivated FBS, 1% penicillin/streptomycin, 1% L-glutamine, and 1% non-essential amino acids. Caco-2 cells were routinely grown at 37 °C in a humidified 5% CO_2_ atmosphere. Media was changed twice per week and cells were passaged at 80% of confluence (cell density at 8 × 10^4^∼1 × 10^5^ cells *per* cm^2^), being harvested by trypsinization (1% trypsin-EDTA solution).

#### Cytotoxicity assays

Cytotoxicity assays were carried out following a previously reported procedure based on the MTT method^15^. MIL-100(Fe) and CS-coated MIL-100(Fe) suspensions (200–1200 μg mL^−1^) were incubated for 24 hours with adherent Caco-2 cells seeded 48 h prior to the assay (1 × 10^4^ cells *per* well in supplemented DMEM).

#### Transepithelial electrical resistance (TEER)

Caco-2 cells were seeded on tissue culture polycarbonate membrane fiaco-2 (0.4 μm pore size, 4.67 cm^2^ growth area) in 6-well Transwell plates at a seeding density of 25 × 10^4^ cells/insert. The culture medium was added to the donor and the acceptor compartments (1.5 mL *per* well in the apical side, together with 2.5 mL at the basolateral compartment). Medium was changed every two days. After 21 days in culture, a cell monolayer was obtained and used in the following assays. The integrity of the monolayers was monitored by measuring the transepithelial electrical resistance (TEER) before and after each experiment at 37 °C, using a Millicell ERS-2 meter. The results are expressed in terms of specific permeability (Ω/cm^2^).

The apical-to-basolateral permeability of MIL-100(Fe) and CS-coated MIL-100(Fe) NPs across monolayers was evaluated. For this purpose, prior to the experiment, the cells were pre-equilibrated for 1 h with fresh transport medium (HBSS) at pH 7.4 in both compartments. After removing the medium, the cells were incubated with uncoated and coated NPs, adding 1.5 mL of each formulation (at a concentration of 620 μg·mL^−1^ in HBSS) in the apical side. In the control wells, the same media (HBSS and DMEM) without NPs were used. At set time points (every 30 min up to 2.5 h), 1 mL of the medium was collected from both sides of the chamber and replaced with fresh medium. After 2.5 up to 24 h, the cells were carefully washed twice with HBSS and replaced with fresh cell culture media, collecting in the last set time the entire medium in the apical and basolateral compartments. TEER values exceeded that period (up to 25 h) to assure the recovery of the monolayer. Finally, the quantification of the linker of the NPs in the basolateral compartment was analyzed by HPLC (see methods), whereas the NP internalization into the cell monolayers after the incubation times were examined by confocal fluorescence microscopy, described in the section below. Each experiment was performed in duplicate^42^,^49^,^50^.

#### Cellular penetration assays by confocal fluorescence microscopy

The performance of CS-coated MIL-100(Fe) NPs into Caco-2 cell monolayers during the transport studies (TEER) was monitored by confocal laser scanning microscopy, as previously reported in case of the MIL-100(Fe) uptake^15^,^38^. Adherent Caco-2 cells were seeded (at density of 25·10^4^ cells *per* well) from cover glasses (24 mm) onto 6-Transwell tissue culture plates. Following the TEER protocol performed, the cells were washed with HBSS and incubated with uncoated and CS_MIL-100(Fe) NPs (at concentration of 640 μg·mL^−1^ *per* well) during the desired incubation times (0.5, 2.5 and 24 h) first in HBSS solution until 2.5 h and changing to fresh culture media (supplemented DMEM) up to 24 h. Once the transepithelial resistance was measured, the cell monolayers were extensively washed with PBS to remove the excess of non-internalized NPs, and then fixed with 4% *p*-formaldehyde for 10 min. For immunofluorescence microscopy (Leica AOBS-SP5 spectral confocal microscope mounted on DMI 6000B inverted microscope, Ar laser excitation), the nuclei of cell monolayers were stained with DAPI (1:100 in PBS, 5 min) mounting the coverslips with Mowiol Fluorescent medium. The cell nucleus and Fe self-reflection of the NPs were observed upon 405/397 and 488/409 nm (λ_Ex_/_Em_), respectively (Fig. 5), using the Leica Application Suite 1.7.0 built 1240, LAS AF software to analyze the images.

#### Complement Activation

The influence of the NPs on the degradation of the complement factor (C3) was evaluated by western blot with a monoclonal anti-C3 antibody (mAb) as previously described^15^. Thanks to the Institutional ethics approval, issued by the Ethics Committee for Clinical Research (Xunta de Galicia, Spain. 2013/272), a pool of human sera from healthy donors was included in the study under their written informed consent. All methods were performed in accordance with relevant guidelines and regulation. These human serum samples were incubated with two different concentrations of MIL-100(Fe) and CS_MIL-100(Fe) NPs (25 and 250 μg·mL^−1^), considering as positive and negative controls, the Zymosan and the PBS respectively. SPSS 15.0 software was used for statistical analysis, where the two-tailed Kolmogorov-Smirnov tests were used for testing normality, considering the data with P values higher than 0.05 as normally distributed. Kruskal-Wallis H test was used to compare non-normally distributed data from two or more groups (coated and uncoated NPs *vs*. negative control). If the Kolmogorov-Smirnov test showed P > 0.05, the Mann-Whitney non-parametric U test was used for continuous data. Results were considered statistically significant at P < 0.05. All data were expressed as means ± standard errors of means.

#### Cytokines evaluation

Cytokine production of human peripheral blood mononuclear cells (PBMCs) in contact with 25 and 250 μg·mL^−1^ of uncoated or CS-coated NPs was evaluated as previously reported^15^ by using the FlowCytomix kit (Human Th1/Th2 11plex Ready-to-Use FlowCytomix Multiplex, eBioscience, Affimetrix and a flow cytometer (FC500, Beckman-Coulter; Miami, FL). Positive (LPS) and negative (PBS alone) controls were included for comparison^15^.

#### Human plasma protein interaction tests

The potential interaction of the human plasma proteins with the MIL-100(Fe) and CS_MIL-100(Fe) NPs (25 and 250 μg·mL^−1^), was investigated as previously reported^15^.

## Additional Information

**How to cite this article:** Hidalgo, T. *et al*. Chitosan-coated mesoporous MIL-100(Fe) nanoparticles as improved bio-compatible oral nanocarriers. *Sci. Rep.* **7**, 43099; doi: 10.1038/srep43099 (2017).

**Publisher's note:** Springer Nature remains neutral with regard to jurisdictional claims in published maps and institutional affiliations.

## Supplementary Material

Supplementary Information

## Figures and Tables

**Table 1:** Main physicochemical characterization for uncoated and CS-coated NPs.

	Medium	MIL-100(Fe)	CS_MIL-100(Fe)
**BET surface area (m^2^g^−1^)**		1570	1590^*^
**Size (nm)**	**Water**	135 ± 20	204 ± 32
	**PBS**	155 ± 61	182 ± 50
	***lis*-SIF**	147 ± 54	175 ± 30
	***lis*-SIF-panc**	168 ± 45	908 ± 36
	***lis*-SIF-muc**	254 ± 40	478 ± 85
	**DMEM**	146 ± 37	273 ± 21
	**HBSS**	163 ± 30	165 ± 5
**ζ-potential (mV)**	**Water**	−18 ± 5	+38 ± 5
	**PBS**	−31 ± 0	−28 ± 0
	***lis*-SIF**	−51 ± 1	−5 ± 3
	***lis*-SIF-panc**	−7 ± 2	−6 ± 3
	***lis*-SIF-muc**	−6 ± 1	−13 ± 9
	**DMEM**	−8 ± 2	−24 ± 0
	**HBSS**	−18 ± 1	+18 ± 2

For all media, both size and ζ-potential are measured in 5 min-aged suspensions to ensure comparable situations.

^*^BET surface area weight corrected by considering the presence of CS in the sample.
