# Supplementary material for: Chitosan-coated mesoporous MIL-100(Fe) nanoparticles as improved bio-compatible oral nanocarriers
Source: Sci Rep. 2017 Mar 3;7:43099. doi: 10.1038/srep43099 (PMC5335263; doi:10.1038/srep43099)
Supplement: Supplementary Information [file srep43099-s1.pdf]

Supporting information

Chitosan-coated mesoporous MIL-100(Fe) nanoparticles as improved bio-compatible oral nanocarriers

T. Hidalgo[1,+], M. Giménez-Marqués[1,+], E. Bellido[1], J. Avila[2], M.C. Asensio[2], F. Salles[3], M.V. Lozano[1], M. Guillevic[1], R. Simon-Vazquez[4], A. Gonzalez-Fernandez[4], C. Serre[1], M.J. Alonso[5] and P. Horcajada[1,6*]

[1]Institut Lavoisier, CNRS UMR 8180, Université de Versailles Saint-Quentin-en-Yvelines, 45 Av. des Etats-Unis, 78035 Versailles cedex, France. [2]Synchrotron SOLEIL & Université Paris-Saclay (L'Orme des Merisiers, Saint-Aubin - BP48, 91192 GIF-sur-Yvette, France. [3]ICGM - UMR5253- Equipe AIME, Université Montpellier II, 2 Place Eugène Bataillon - CC 1502, 34095 Montpellier CEDEX 5, France. [4]Immunology, Biomedical Research Center (CINBIO) and Institute of Biomedical Research of Vigo (IBIV), Universidad de Vigo, Campus Lagoas Marcosende, 36310 Vigo, Pontevedra. Spain. [5]Nanobiofar. Center for Molecular Medicine and Chronic Diseases (CIMUS), Universidad de Santiago de Compostela, Av. Barcelona s/n, Campus Vida, 15706 Santiago de Compostela, Spain. [6]IMDEA Energy. Av. Ramón de la Sagra 3, 28935 Móstoles, Madrid, Spain.

[+] Equally contributing authors

[*]patricia.horcajada@imdea.org

Content

Figure S1. TGA.
Figure S2. FTIR and TEM images.
Figure S3. XRPD patterns.
Figure S4. N_2 adsorption isotherm.
Figure S5. Molecular simulation.
Estimation of the polymer conformation and density of coating
Figure S6. Chemical structure of chitin.
Table S1. Composition of intestinal simulated media (SIF)
Figure S7. Colloidal stability of MIL-100(Fe) and CS_MIL-100(Fe) NPs
Figure S8. Cytotoxicity assays. Cell viability of Caco-2 cell line after 24 h.
Figure S9. Measurements of protein conformational changes.
Figure S10. Cell uptake studies. Confocal microscopy images after 0.5 h.
Figure S11. Cell uptake studies. Confocal microscopy images after 2.5 h.

Figure S12. Cell uptake studies. Confocal microscopy images after 24 h.

Figure S13. Transepithelial resistance assay.

Figure S14. Top: Complement activation studies.

Figure S15. Human cytokine production.

Table S2. Summary of the cytokine production.

Table S3. Quantification of the NPs crossing by HPLC.

References

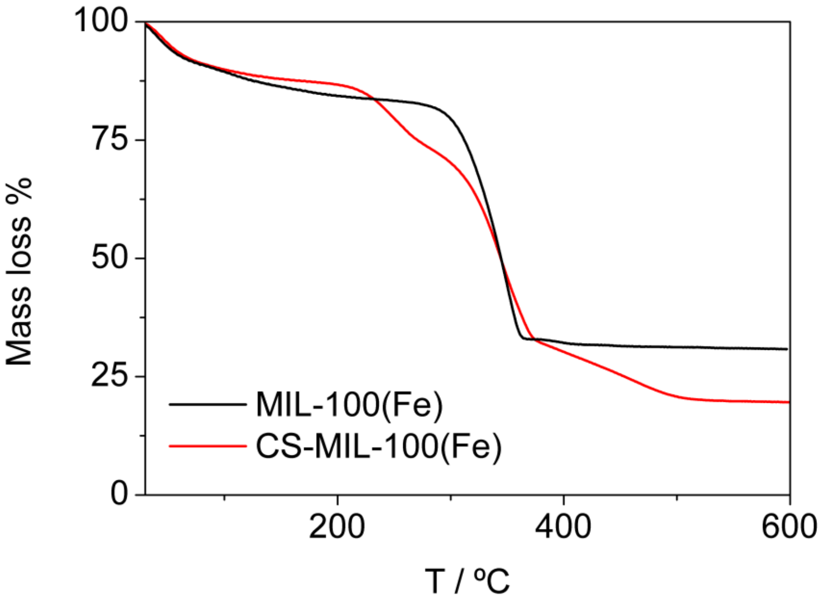

Figure S1. TGA of MIL-100(Fe) NPs (black) and CS_MIL-100(Fe) NPs (red line).

The extent of CS coating on MIL-100(Fe) NPs was quantified by fluorescence spectrometry and TGA. The amount of CS coating on the NPs was indirectly estimated by the difference between the CS concentration in the initial reaction mixture (2.50 ± 0.01 $mg \cdot mL^{-1}$) and the supernatant collected after reaction and subsequent washing steps (0.14 ± 0.03 $mg \cdot mL^{-1}$). These values conclude that 49.1 ± 0.3 wt % of the final coated CS_MIL-100(Fe) NPs corresponds to CS coating. Consequently, a highly efficient CS grafting of 94.4 ± 0.4 % was obtained in only 30 min. of reaction (considering as 100 % the total amount of CS in the initial impregnation conditions).

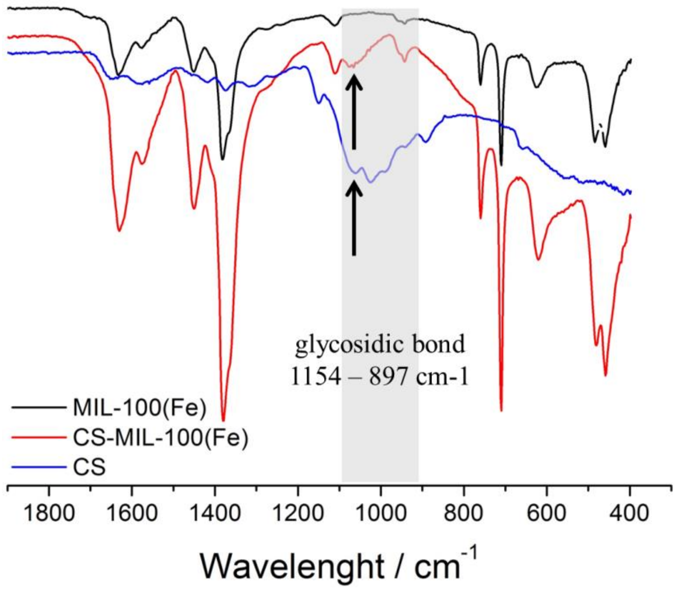

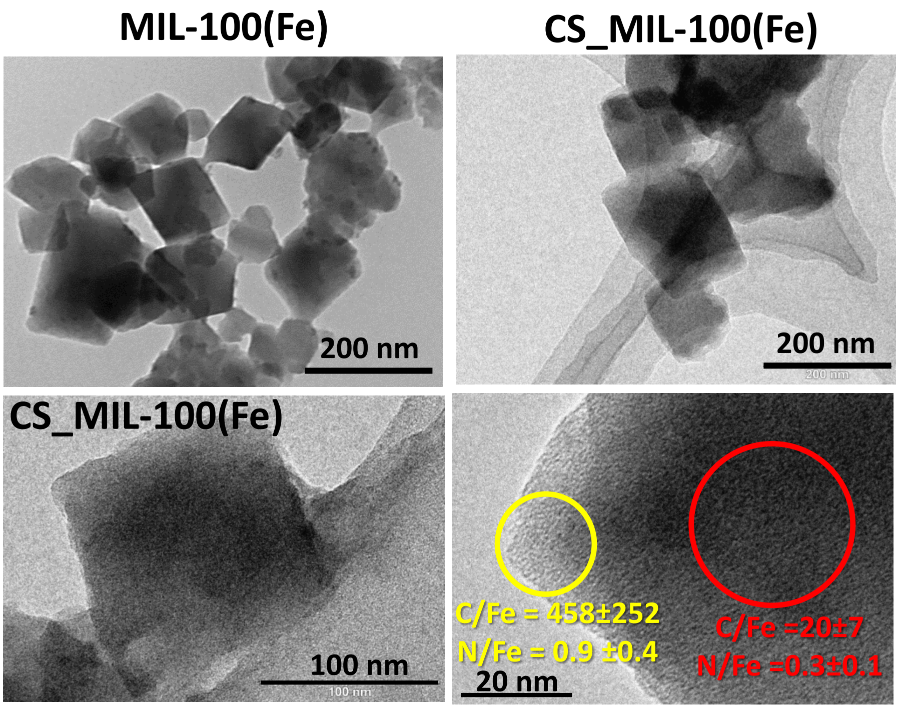

Figure S2. Top: FTIR spectra of CS_MIL-100(Fe) NPs (red) in comparison with the uncoated (black) and chitosan (blue line). Center: TEM images of MIL-100(Fe) and CS_MIL-100(Fe) NPs. Bottom: TEM images of CS_MIL-100(Fe) NPs together with the C/Fe and C/N ratio of the layer and bulk sections of the NPs (from EDX analysis).

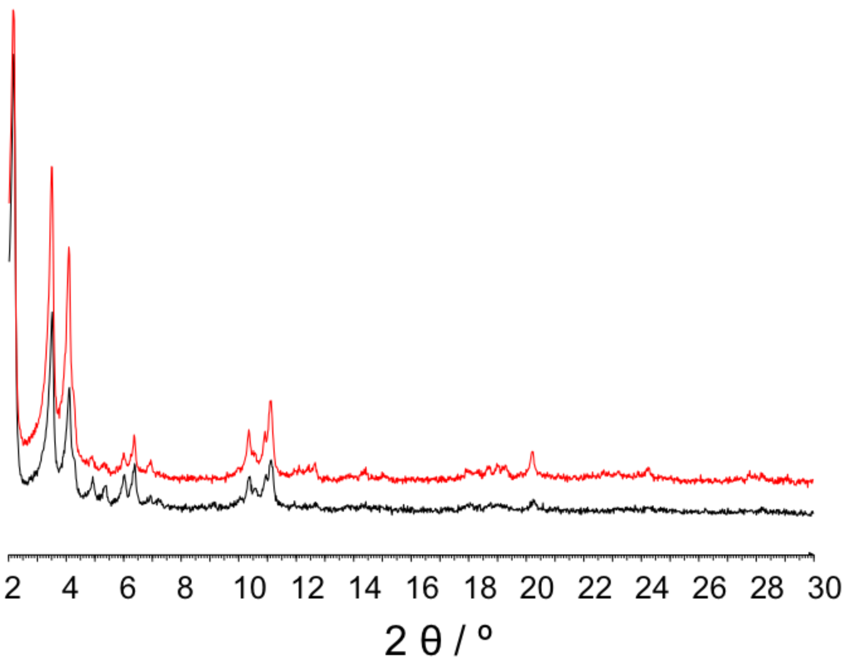

Figure S3. XRPD patterns of MIL-100(Fe) nanoparticles before (black) and after (red) external surface modification with chitosan (XRPD collected in a D8 Advance Bruker diffractometer with Cu Kα1 radiation (λ = 1.54056 Å) from 3 to 30º (2θ) using a step size of 0.02º and 2.5 s per step in continuous mode).

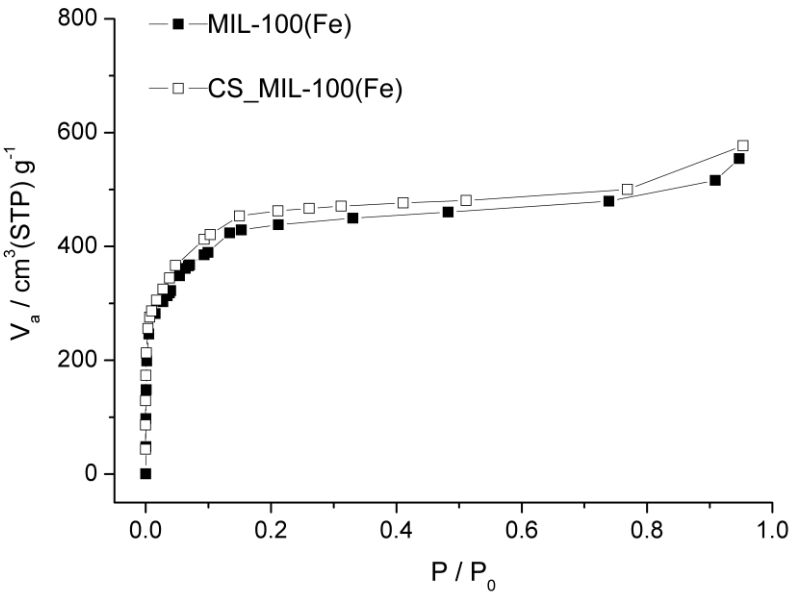

Figure S4. N_2 adsorption isotherm at 77 K of MIL-100(Fe) NPs before (full squares) and after CS-coating (empty squares) after outgassing at 140 °C for 3 h. (note: weight correction is applied for CS_MIL-100(Fe) NPs).

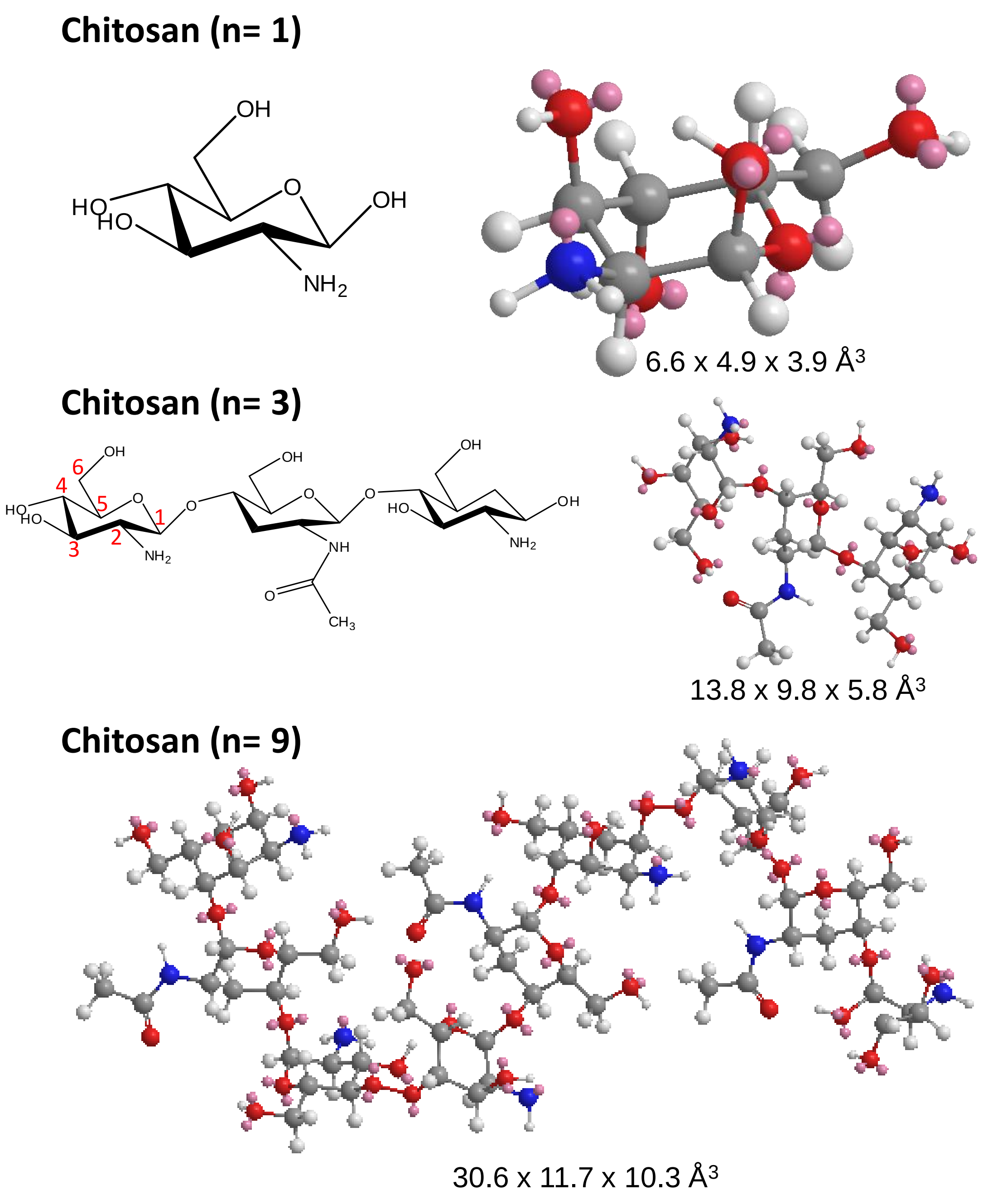

Figure S5. Molecular simulation of CS (above) as three- (middle) and nine-units (below) oligomers. The size of the molecule is indicated for three different orientations.

Estimation of the polymer conformation and density of coating

One can estimate the polymer density using the conformation of the polymer chains on the NP external surface, which is mainly determined by: (i) the distance between grafted CS chains (D) (ii) the length/thickness (*L*) of the grafted CS layer and (ii) the Flory radius of the grafted CS (R_F).[1]

Flory radius R_F: Polymer conformation can be estimated in terms of the Flory radius (R_F), which is the average coil dimension in solution, and the distance between grafted chains (D).

Considering these parameters, there are two main conformations: if the surface density is low ($D > R_F$) the CS chains adopt a "mushroom" conformation and remain not fully extended away from the NP surface. In case of increased coating densities ($D < R_F$), the CS chains acquire a "brush" conformation with a thick layer of CS chains extended away from the NP surface.

If the solution is a good solvent for the polymer (i.e. CS in water:ethanol) the Flory radius can be described according to the following equation: $R_F = \alpha \cdot n^{3/5}$, where α is the length of one CS monomer, and n is the number of monomers *per* polymer chain. In our case, we have estimated the conformation of CS chains considering the following parameters: (i) the thickness of the coating CS layer (~ 35 nm, as determined by DLS measurements), (ii) the length of a single monomer of CS, taking as the monomer 9 units linked through β-(1-4) bridges: 6 units of D-glucosamine and 3 of N-Acetyl-D-glucosamine (~ 30.6 Å, as determined by molecular simulation under vacuum, Figure S5) and (iii) the length of CS chains (MW ~ 50000 Da, *n* ~ 30 monomers *per* chain, extended CS length ~ 90 nm).

Then, the calculated Flory radius is $R_F \sim 23$ nm.

The calculated distance between grafted CS chains is D ~ 3 nm, whereas the Flory radius is $R_F \sim$ 23 nm.

Thus, as the distance between CS chains is lower than the Flory radius (D ~ 3 nm < $R_F \sim$ 23 nm) and the thickness of the grafted CS layer (L) is of ~ 35 nm (L < calculated length of a CS chain), CS chains are expected to follow a "brush" conformation with partial folding of the polymer. One could consider that such a conformation of the CS coating may be beneficial to the interaction of the NPs with biological gastrointestinal structures (mucus, enzyme, enterocytes, etc) modifying its oral stability and absorption.

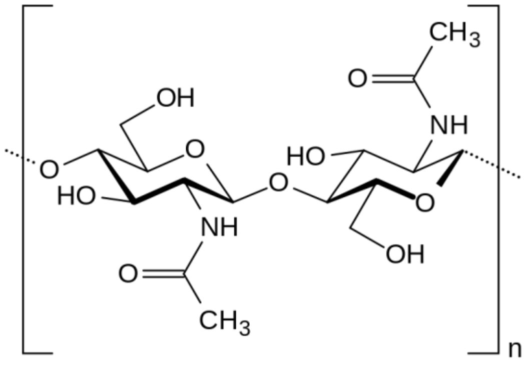

Figure S6. Chemical structure of chitin.

Table S1. Composition of intestinal simulated media: SIF – lis-SIF, lis.

**Composition**	**SIF**		**Supplemented *lis*-SIF**	
	**Conc/mM**	**g *per* 500 mL**	**Conc/mM**	**g *per* 500 mL**
Sodium hydroxide	15.4	0.300	0.6	0.012
Monobasic potassium phosphate	50	3.400	2.0	0.136
**Pancreatin**	---	---	---	5
**Mucin**	---	---	---	25
pH	6.8		6.8	

- Simulated intestinal fluid (SIF) was prepared as follows : 38.5 mL of a 0.2 M NaOH solution were added to a solution of 3.4 g of KH_2PO_4 dissolved in 125 mL of Milli-Q water.[2] Then, Milli-Q water was added until a volume of 500 mL to finally adjust the pH to 6.8 with 2 M NaOH.

- *lis*-SIF: Low-ionic-strength SIF corresponds to a 1/25 diluted SIF.

- *lis*-SIF-panc: *lis*-SIF supplemented with pancreatin was prepared by dissolving the pancreatin at 1 % w/v in *lis*-SIF and stirring the mixture for 3 h. Then, the solution was centrifuged (10500 rpm, 10 min) to eliminate pancreatin aggregates. Note that the final pancreatin concentration of 1% is rather an estimated value.

- *lis*-SIF-muc: *lis*-SIF supplemented with mucin was prepared by dissolving the mucin at 5 % w/v in in *lis*-SIF,[3] keeping the mixture under magnetic stirring for 3 h 30 min.

Prior to the analysis, the biological media were kept at 37 °C.

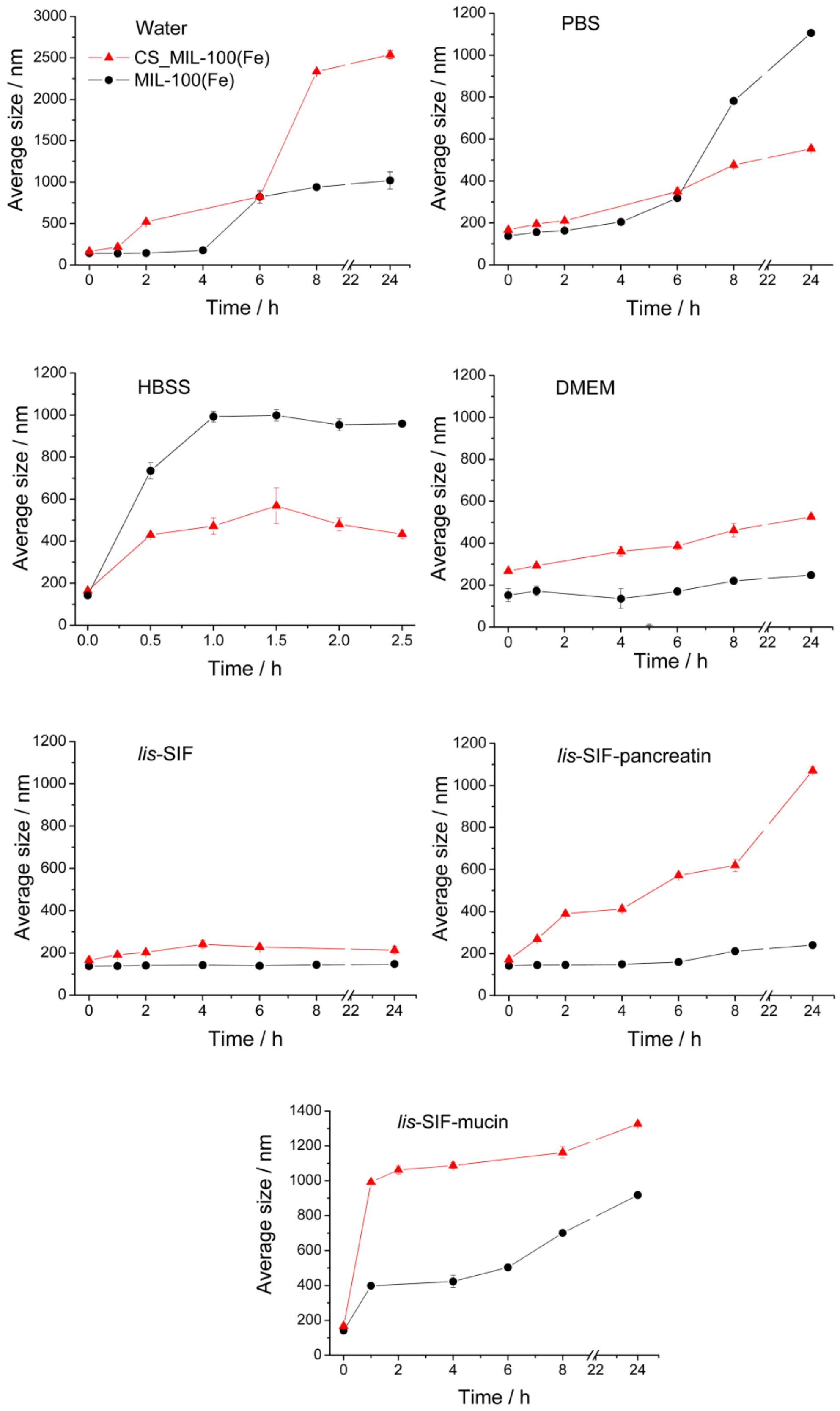

Figure S7. Colloidal stability of MIL-100(Fe) (black) and CS_MIL-100(Fe) NPs (red) in: i) water, ii) PBS, iii) HBSS, iv) DMEM, v) *lis*-SIF, vi) *lis*-SIF-panc and vii) *lis*-SIF-mucin, representing the NP size evolution *vs.* Time.

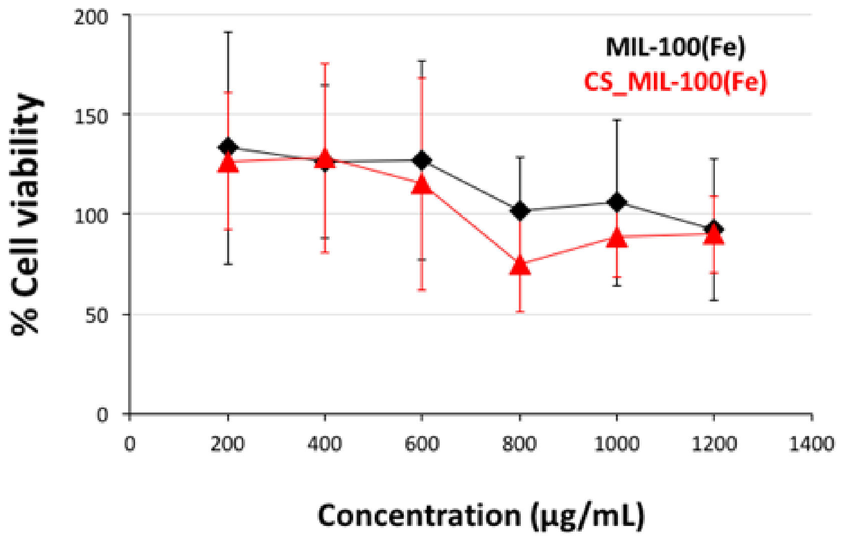

Figure S8. Cell viability of Caco-2 cell line after 24 h in contact with uncoated or CS_MIL-100(Fe) NPs. Note that these data shown in each concentration, corresponding to the average of the quadruplicate replicate obtained in three independent experiments (n=12). The vertical error bars drawn in the diagram indicate the range of fluctuations from which the standard deviations were calculated.

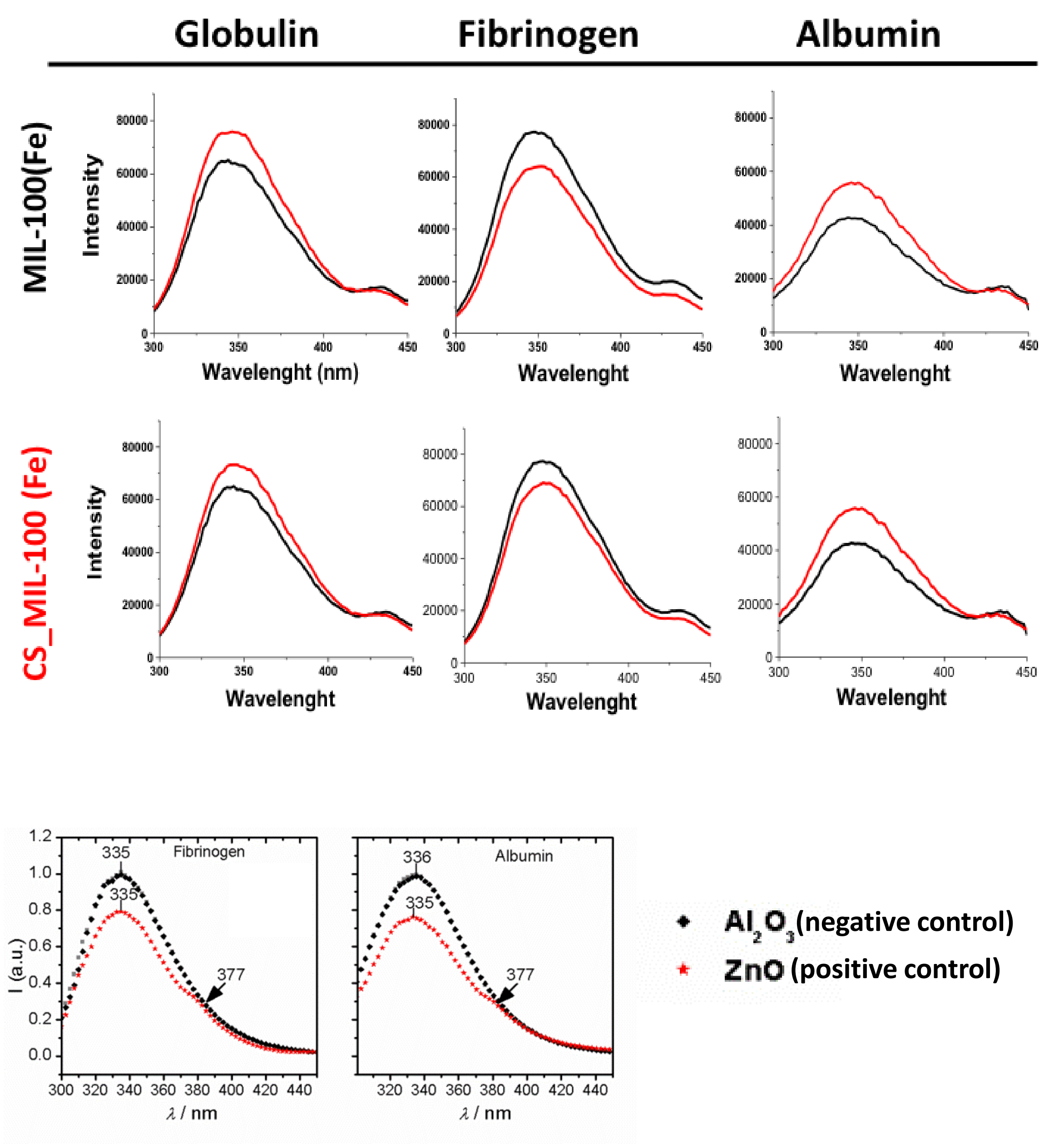

Figure S9. Top: Tryptophan fluorescence spectroscopy of different plasmatic protein fractions in contact with MIL-100(Fe) and CS_MIL-100(Fe) NPs (in red: 100 µg of NPs *per* 440 µg of globulin *per* mL (left); 100 µg of NPs *per* 160 µg of fibrinogen *per* mL (center); 100 µg of NPs *per* 1000 µg of albumin *per* mL (right)). An identical control in absence of NPs is represented in black for comparison. These data correspond to the average of two replicates obtained in two independent experiments (n = 4). **Bottom**: Tryptophan fluorescence spectroscopy of fibrinogen and albumin fractions in contact with Al_2O_3 NPs and ZnO NPs considered as negative and positive controls, respectively.

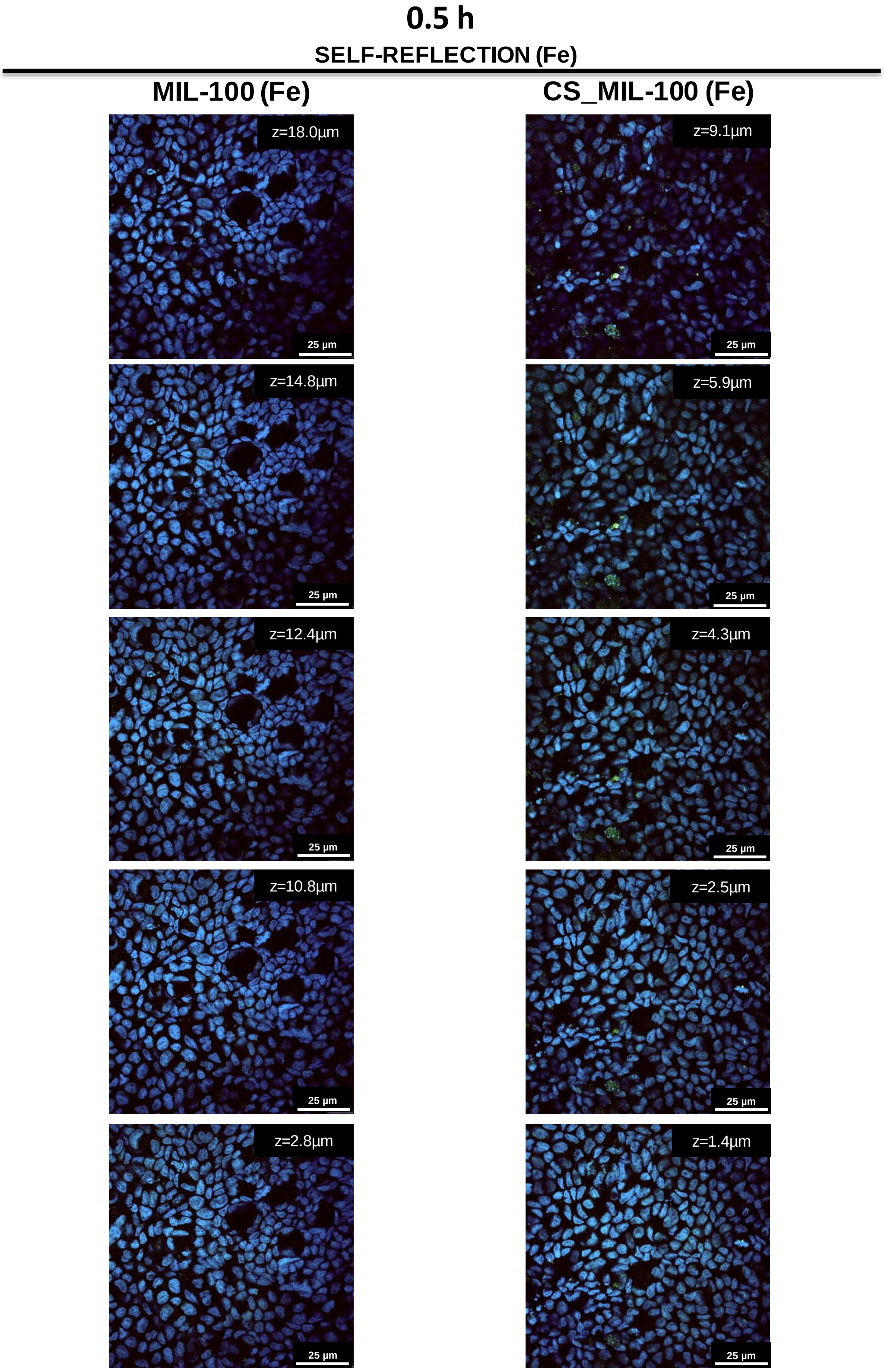

Figure S10. Confocal microscopy images at different depths in the Z-axis of Caco-2 cell monolayer containing uncoated and chitosan coated MIL-100(Fe) NPs observed by iron self-reflection signal (in green) and the nucleus stained with DAPI (in blue) after 0.5 h. The scale bar corresponds to 25 µm. All the images are taken at 63X.

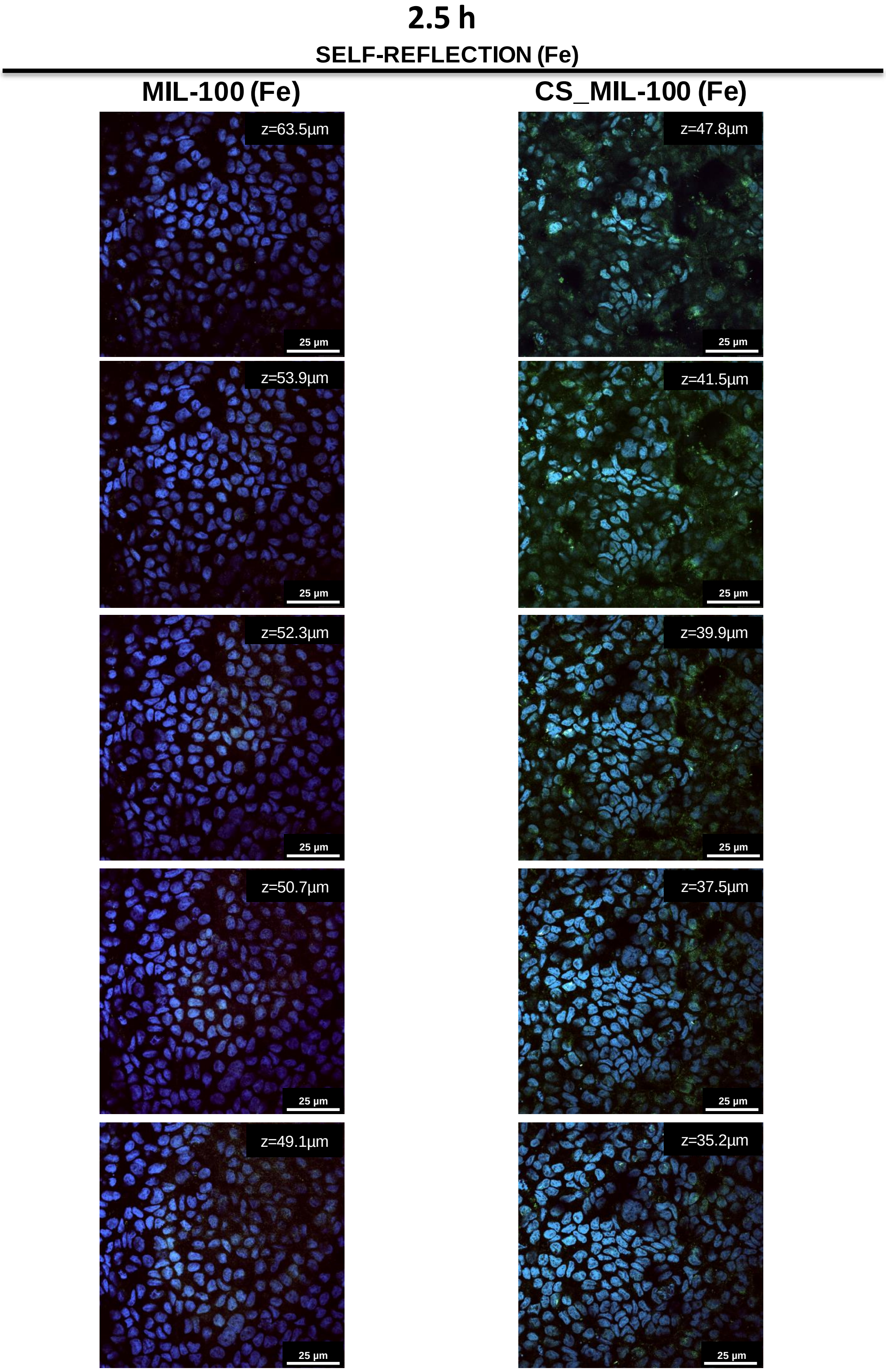

Figure S11. Confocal microscopy images at different depths in the Z-axis of Caco-2 cell monolayer containing uncoated and chitosan coated MIL-100(Fe) NPs observed by iron self-reflection signal (in green) and the nucleus stained with DAPI (in blue) after 2.5 h. The scale bar corresponds to 25 µm. All the images are taken at 63X.

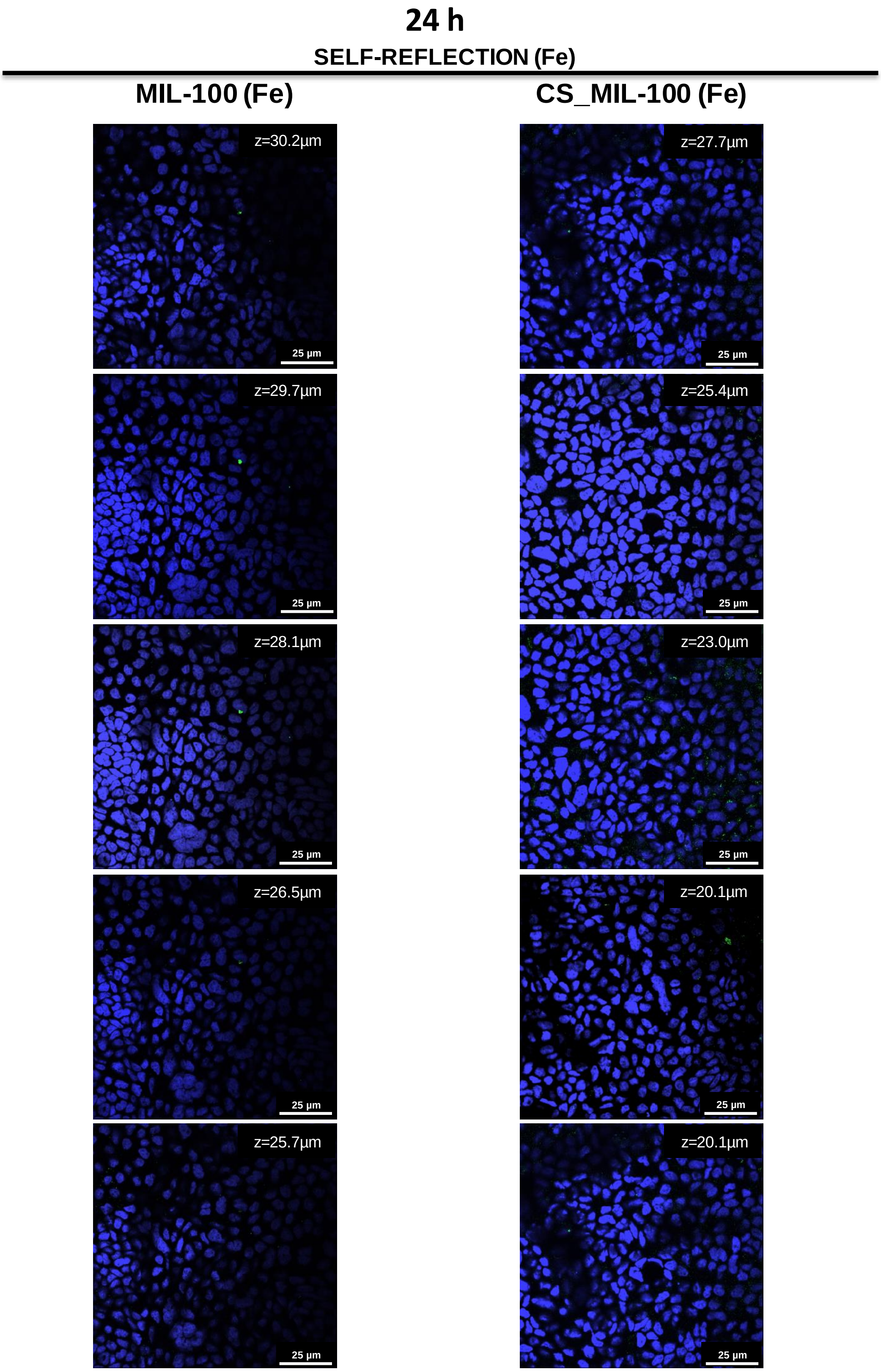

Figure S12. Confocal microscopy images at different depths in the Z-axis of Caco-2 cell monolayer and the nucleus stained with DAPI (in blue) after 24 h. The scale bar corresponds to 25 µm. All the images are taken at 63X.

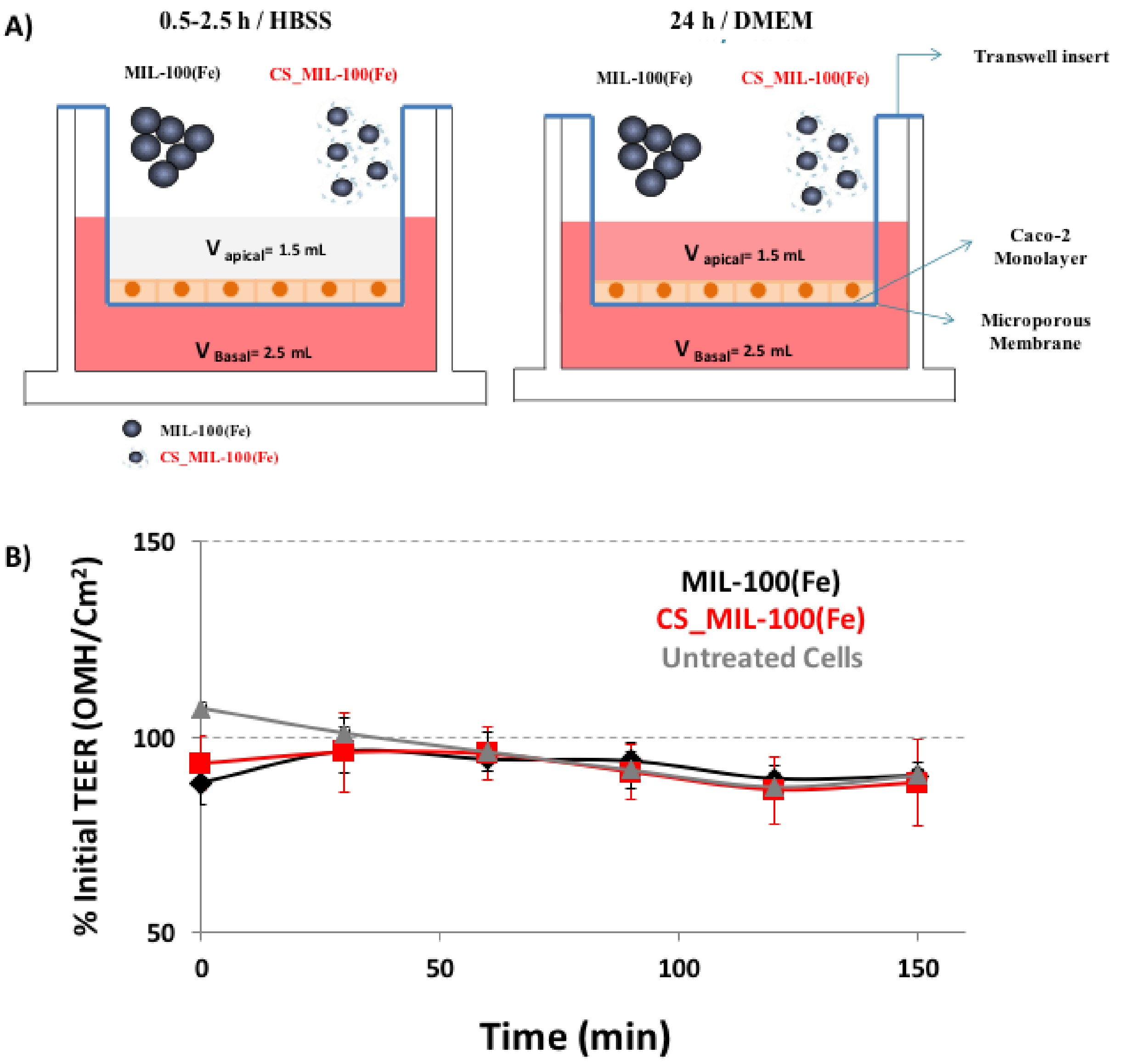

Figure S13. A) Scheme of Transepithelial resistance assay developed until 24h. B) Transepithelial resistance measurement provided by Caco-2 cell line after 2.5 h of contact with uncoated and CS_MIL-100(Fe) NPs.

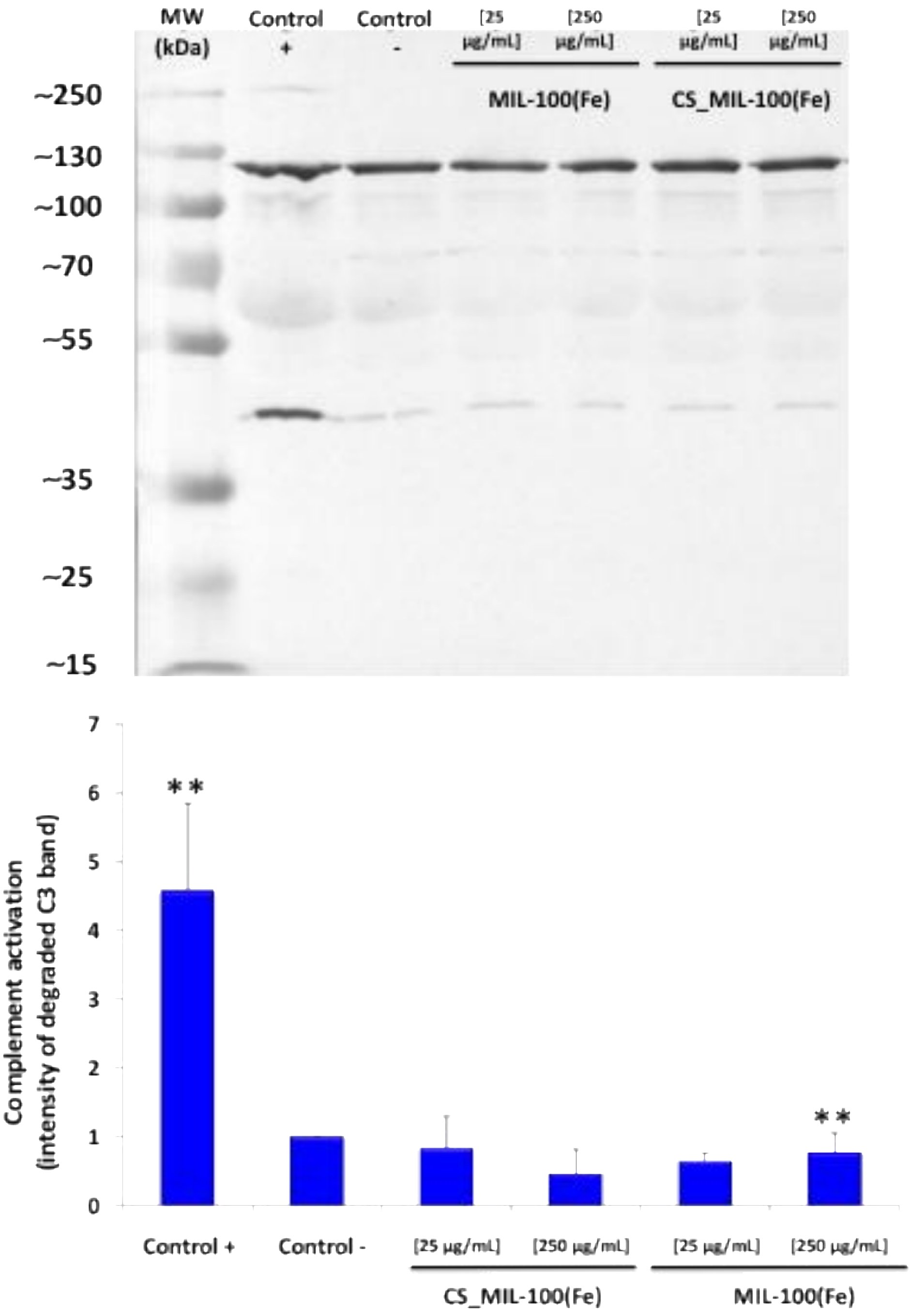

Figure S14. Top: Complement activation was represented by determining the intensity of the band at 43 kD, corresponding to the degraded C3 factor, and compared with the band at 115 kD, corresponding to the intact protein. The samples were normalized respect to the negative control. **Bottom**: Complement activation data for uncoated and CS_MIL-100(Fe) NPs determined by Western blot using a specific C3 antibody to measure the degradation of the protein. Note that these data were obtained by duplicates from three independent experiments (*n*=6) and the error bars correspond to the standard deviation. Statistically significant differences between the control group and the coated/uncoated group were evidenced by the Mann-Whitney U test (P<0,05)**.

Statistical results for all of the groups :

- Negative control – Positive control: 0.02*
- Negative control – MIL-100(Fe) [25 $mg.mL^{-1}$]: 0.059
- Negative control – MIL-100(Fe) [250 $mg.mL^{-1}$]: 0.257
- Negative control – CS_MIL-100(Fe) [25 $mg.mL^{-1}$]: 0.317
- Negative control – CS_MIL-100(Fe) [250 $mg.mL^{-1}$]: 0.023*

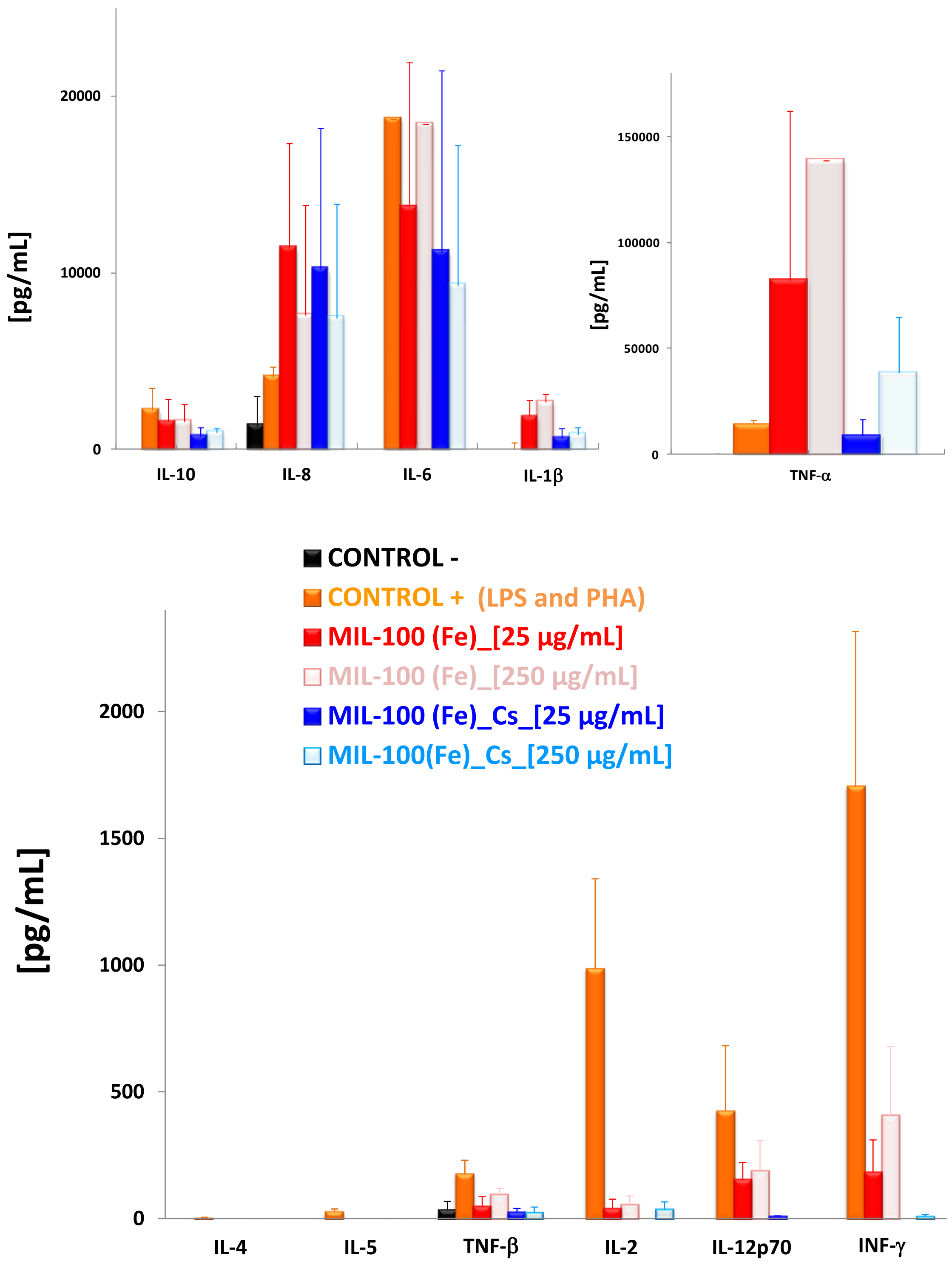

Figure S15. Human cytokine production from peripheral blood mononuclear cells (3 different donors) after 24 h in contact with 25 or 250 μg/mL of MIL-100(Fe) and CS_MIL-100(Fe) NPs.

Table S2. Summary of the cytokine production. The values correspond to the variation of the concentration of cytokines (in pg·mL^{-1}) in comparison with the negative control (<1, 1-10, 10^2-10^3, 10^3-10^4 or >10^4 times) obtained from 3 different donors (1/3, 2/3 or 3/3), with the representation of the activation showed in the positive control (LPS and PHA).[4]

		**NPs dose (µg/mL)**	**LPS and PHA**	**MIL-100(Fe)**	**CS_MIL-100(Fe)**
**Th1 cytokines**	IL-12p70	25	**3/3** (10^2-10^3)	**3/3** (10^2-10^3)	**2/3** (1-10)
		250			**3/3** (0)
	INF-γ	25	**3/3** (10^3-10^4)	**2/3** (10^2-10^3)	**2/3** (0-1)
		250		**3/3** (10^2-10^3)	**2/3** (1-10)
	IL-2	25	**2/3** (10^2-10^3)	**2/3** (10-10^2)	**2/3** (0-1)
		250		**3/3** (10-10^2)	**2/3** (10-10^2)
**Th2 cytokines**	IL-10	25	**2/3** (10^3-10^4)	**2/3** (10^3-10^4)	**2/3** (10^2-10^3)
		250			**2/3** (10^3-10^4)
	IL-6	25	**3/3** (>10^5)	**3/3** (10^2-10^3)	**2/3** (10^3-10^4)
		250			
**Other proinflammatory cytokines**	IL-8	25	**2/3** (10^3-10^4)	**2/3** (10^2-10^3)	**2/3** (10^4-10^5)
		250		**3/3** (10^2-10^3)	**2/3** (10^3-10^4)
	IL-1β	25	**3/3** (10^2-10^3)	**3/3** (10^3-10^4)	**2/3** (10^2-10^3)
		250			**3/3** (10^2-10^3)
	TNF-α	25	**3/3** (10^3-10^4)	**2/3** (>10^5)	**2/3** (10^4-10^5)
		250		**3/3** (>10^5)	**3/3** (10^4-10^5)
	TNF-β	25	**3/3** (10^3-10^4)	**2/3** (10^2-10^3)	**2/3** (10-10^2)
		250		**3/3** (10^2-10^3)	

*IL-4 and IL-5 secretion was not observed with both MIL-100(Fe) and CS_MIL-100(Fe) NPs.

Table S3. Quantification of the NPs crossing by HPLC

	**% NPs in basolateral compartment**		
**Contact time**	**0.5h**	**2.5h**	**24h**
**MIL-100(Fe)**	0.30 ± 0.02	0.10 ± 0.04	0.20 ± 0.09
**CS_MIL-100(Fe)**	0.44 ± 0.03	0.50 ± 0.07	4.10 ± 0.03

References

[1] (a) De gennes, P. G. Conformations of Polymers Attached to an Interface. *Macromolecules* **13**, 1069-1075 (1980). (b) Jokerst, J. V., Lobovkina, T., Zare, R. R. & Gambhir, S. S. Nanoparticle PEGylation for imaging and therapy. *Nanomedicine* **6**, 715-728 (2011).

[2] European Pharmacopoeia 2011 (7th Edition). Council of Europe; European Directorate for the Quality of Medicine, Strasbourg.

[3] Lai, S. K., Wang, Y.Y., Wirtz, D. & Hanes, J. Micro- and macrorheology of mucus *Adv. Drug Deliv. Rev*. **61**, 86-100 (2009).

[4] Simón-Vazquez, R., Lozano-Fernández, T., Davila-Grana, A. & González-Fernández, A. Analysis of the activation routes induced by different metal oxide nanoparticles on human lung epithelial cells. *Futur. Sci. OA*, 2(2), FSO118, (2016).
